# Screening and Development of New Inhibitors of FtsZ from *M*. *Tuberculosis*

**DOI:** 10.1371/journal.pone.0164100

**Published:** 2016-10-21

**Authors:** Bini Mathew, Judith Varady Hobrath, Larry Ross, Michele C. Connelly, Hava Lofton, Malini Rajagopalan, R. Kiplin Guy, Robert C. Reynolds

**Affiliations:** 1 Department of Chemistry, The University of Alabama at Birmingham, Birmingham, Alabama, 35294, United States of America; 2 Division of Hematology and Oncology, The University of Alabama at Birmingham, Birmingham, Alabama, 35294, United States of America; 3 Drug Discovery Division, Southern Research Institute, 2000 Ninth Avenue South, Birmingham, Alabama, 35205, United States of America; 4 Drug Discovery Unit, College of Life Sciences, University of Dundee, Dundee, DD1 5EH, United Kingdom; 5 Dept. Chemical Biology & Therapeutics, St Jude Children's Research Hospital, 262 Danny Thomas Place, Memphis, TN, 38105, United States of America; 6 The University of Texas Health Science Center at Tyler, Tyler, Texas, 75708, United States of America; Rutgers Biomedical and Health Sciences, UNITED STATES

## Abstract

A variety of commercial analogs and a newer series of Sulindac derivatives were screened for inhibition of *M*. *tuberculosis (Mtb) in vitro* and specifically as inhibitors of the essential mycobacterial tubulin homolog, FtsZ. Due to the ease of preparing diverse analogs and a favorable *in vivo* pharmacokinetic and toxicity profile of a representative analog, the Sulindac scaffold may be useful for further development against *Mtb* with respect to *in vitro* bacterial growth inhibition and selective activity for *Mtb* FtsZ versus mammalian tubulin. Further discovery efforts will require separating reported mammalian cell activity from both antibacterial activity and inhibition of *Mtb* FtsZ. Modeling studies suggest that these analogs bind in a specific region of the *Mtb* FtsZ polymer that differs from human tubulin and, in combination with a pharmacophore model presented herein, future hybrid analogs of the reported active molecules that more efficiently bind in this pocket may improve antibacterial activity while improving other drug characteristics.

## Introduction

Tuberculosis (TB), caused by *Mycobacterium tuberculosis* (*Mtb*), is one of the most prevalent infectious diseases worldwide. A recent report by the World Health Organization (WHO) reveals that in 2014 there were an estimated 9.6 million new cases of TB (12% co-infected with HIV) and 1.5 million people died from the disease, including 1.1 million deaths among HIV-negative individuals and 400,000 among people who were HIV-positive.[1] Moreover, the increasing development of multi-drug resistant TB (MDR-TB) strains, forms of TB that do not respond to standard front line regimens, is a serious burden on public health systems throughout the world. More recently, extensively drug resistant tuberculosis (XDR-TB) that is also resistant to second line drugs has emerged in numerous areas. These stark realities emphasize the acute need for constant reinvention of the anti-TB drug pipeline targeting novel, alternative drug pathways.[2–3]

New targets for tuberculosis are regularly being identified from existing genomic data and new screening techniques.[3] The bacterial tubulin homolog, FtsZ (Filamenting temperature-sensitive protein Z), is essential for bacterial cell division and is an ideal target for novel antimicrobials. In recent years, FtsZ has received considerable attention for the discovery of novel antibacterial and anti-TB drugs.[4–17]

In our search for new active scaffolds against *Mtb* FtsZ, a variety of small molecules reported to have antibacterial activity, and, furthermore, some of which were considered to inhibit *Mtb* or other bacterial FtsZs were acquired and screened for both antitubercular activity and *Mtb* FtsZ inhibition. A consistent set of antibacterial activity data in parallel with FtsZ screening results should be useful to prioritize active scaffolds for new analog optimization. Furthermore, the potent combination of a new crystal structure and these activity data will allow advancement of robust consensus binding models that should help medicinal chemists enhance selective activity against the bacterial protein target and whole bacteria while potentially minimizing off-target effects against the direct mammalian homolog, tubulin, as well as reducing mammalian toxicity through other off-target activities. Beyond the aforementioned antibacterial/FtsZ actives or related compounds, we were particularly intrigued by the reported similarities of certain non-steroidal anti-inflammatory drugs (NSAIDs), e.g. Indomethacin and Sulindac analogs, to the known tubulin polymerization inhibitor Colchicine.[18,19] Colchicine has been reported to be one of the few known tubulin inhibitors that demonstrates activity against *Mtb* FtsZ.[15] Sulindac belongs to this chemically diverse group and, importantly, is not overtly toxic *in vivo* but shows clinical efficacy for longer term treatment regimens in cancer chemoprevention.[20–23] The NSAIDs are excellent pharmacophores showing good activity through animal models and in the clinic for numerous indications. As part of an ongoing program to study the chemical biology of interesting NSAID scaffolds such as Sulindac, we have investigated a variety of analogs and their on-target (COX-1 and 2) and off-target (e.g. cell cytotoxicity, PDE5, PDE10A) activities.[24–25] Among the interesting and atypical activities of the NSAIDs, certain known drugs (e.g. Ibuprofen, Aspirin) have been reported to show antibacterial activity.[26–30] An indomethacine analog closely related to sulindac sulfide amide (SSA) has been reported to inhibit tubulin polymerization *in vitro* in a dose response manner.[18] Hence, we added this early lead Sulindac analog to our initial anti-TB/FtsZ assays and confirmed that it is a modest potency inhibitor of *Mtb* FtsZ while showing no inhibition of human tubulin at 100 μM concentration. The activity of this initial lead warrants the exploration of new sulindac analog series against FtsZ. Herein, we report the screening of a number of acquired and synthesized samples and a lead Sulindac analog available in our labs against FtsZ from *M*. *tuberculosis*. These compounds advanced in a series of assays depending on activity that include *Mtb* FtsZ, *Mtb* H_37_Ra, MAC NJ211 and/or *Mtb* H_37_Rv, tubulin polymerization, and in a preliminary cell cytotoxicity assay against BJ cells, an immortalized normal human foreskin fibroblast cell line. In addition to the presented structure-activity development of the Sulindac scaffold, we also followed up a potent and previously reported screening hit, Zantrin Z2, which showed potent activity in our preliminary screens (see S1 Appendix in Supporting Information for results).

## Materials and Methods

### Animal ethics statement

All experimental protocols were approved with written consent by the Animal Care and Use Committee of Colorado State University (approval number ACUC no. 12-3723A), which abides by the USDA Animal Welfare Act and the Public Health Service Policy on Humane Care and Use of Laboratory Animals.

### Animal care and euthanasia

The CSU animal assurance welfare number is A3572-01 under file with NIH. All animals are cared for by the Colorado State Lab Animal Resources, headed by two experienced veterinarians and a large number of support staff. The mice are observed twice daily by our Research Associates and Lab Animal Resources (LAR) personnel. A log is kept recording any untoward behavior. Sick animals are reported to the Staff Veterinarian on a morbidity form. Investigators receive a copy of the morbidity form with initial physical exam findings, diagnostics, and possible diagnoses. The LAR at Colorado State University has procedures in place to control animal pain. Specific health and comfort parameters (activity and temperament, feeding behavior, appearance) are used to monitor pain and severity of disease of M. tuberculosis infected mice, and these conditions are monitored daily to determine the condition of the mice. When issues are evident (distress, severe physiologic changes, weight loss), mice are required to be euthanized within the day. The method used for euthanasia of mice is carbon dioxide inhalation. This method, approved by the CSU Regulatory Compliance Office uses either tank CO2 or house CO2 This method is approved by the AVMA Panel of Euthanasia, and was chosen for its reliability, ease of use, and minimization of distress to animals.

### Chemical synthesis of sulindac analogs

**Fig 1:**
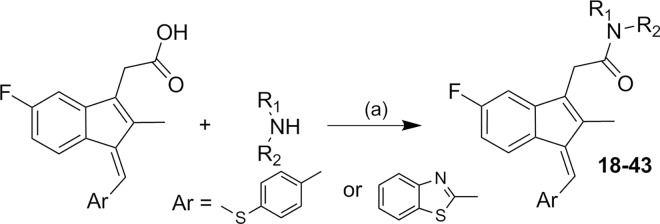
Synthetic pathways to analogs 18–43. Reagents and conditions: (a) HBTU or HATU, TEA or DIEA, MeCN.

Compounds **18–43** were synthesized by the coupling of commercially available Sulindac sulfide or a new 1-benzothiazolyl Sulindac derivative (prepared from 2-(5-fluoro-2-methyl-1H-inden-3-yl)acetic acid and benzothiazole-2-carboxaldehyde according to a literature procedure)[31] with various amines using either HBTU[32] or HATU[33] as the coupling agent (Fig 1).

**Fig 2:**
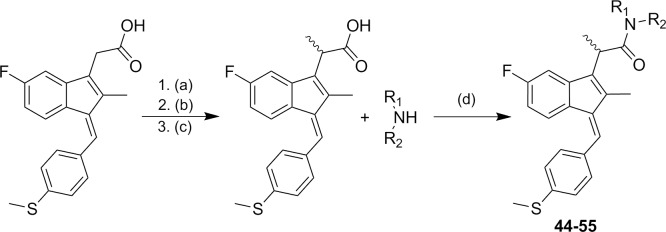
Synthetic pathways to analogs 44–55. Reagents and conditions: (a) MeOH, SOCl_2_ (b) LDA. MeI, THF, −78°C (c) KOH, EtOH/H_2_O (d) HATU, DIEA, MeCN.

Preparation of α-methyl Sulindac sulfide amide began with esterification of commercially available Sulindac sulfide in the presence of MeOH/thionyl chloride to provide Sulindac sulfide methyl ester in quantitative yield. The introduction of a methyl group at the α-position of the ester group was carried out using LDA and CH_3_I to afford α-methyl Sulindac sulfide ester in 93% yield as a mixture of enantiomers.[34] α-Methyl Sulindac sulfide ester was hydrolyzed to give the corresponding acid in excellent yield. Finally, the coupling of α-methyl Sulindac sulfide with various amines in the presence of HATU[33] as the coupling agent afforded compounds **44–55** in good yields (Fig 2). Typically, when the racemic mixture of the acid was treated with a chiral amine, an inseparable mixture of diastereomeric amides was formed.

**Fig 3:**
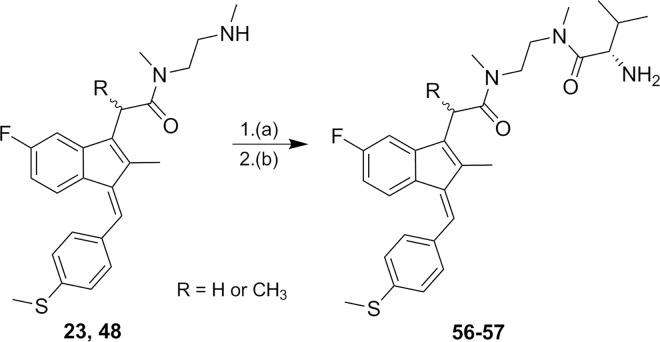
Synthetic pathways to analogs 56–57. Reagents and conditions: (a) HBTU or HATU, TEA or DIEA, MeCN (b) H^+^.

N,N’-Dimethylaminoethyl amides of Sulindac sulfide and α-methyl Sulindac sulfide (**23**, **48**) were treated with Boc-L-Valine under the same coupling reaction conditions described in Fig 1 and Fig 2 followed by treatment with acid for the removal of the Boc protecting group to produce **56–57** in good yields (Fig 3).

**Fig 4:**
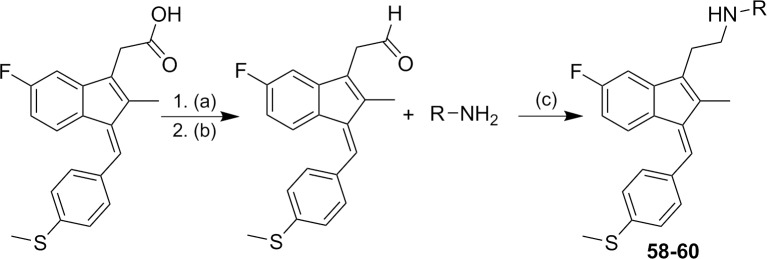
Synthetic pathways to analogs 58–60. Reagents and conditions: (a) MeOH, SOCl_2_ (b) DIBALH, Toluene (c) NaBH_4_, MeOH, rt.

The synthetic strategy used to prepare the target compounds **58–60** is outlined in Fig 4. Synthesis began with the esterification of Sulindac sulfide to form its methyl ester. The ester was then converted to its corresponding aldehyde by treating with DIBALH. Finally, reductive amination of the aldehyde with various substituted amines afforded compounds **58–60** in moderate yields.[35]

Compounds **61–63** were prepared by methods given above (Fig 1).[31, 32] Further synthetic experimental details are provided in S2 Appendix under Supporting information.

#### Compounds

All compounds were purchased commercially (Aldrich, ChemBridge, or CalBiochem) and used as provided excepting the Sulindac analogs that were prepared as described earlier (see also Supporting Information). Sanguinarine and Pseudojatorrhizine were provided by the University of Mississippi National Center for Natural Product Research, and Totarol was a kind gift of Industrial Research Limited (New Zealand).

### Biological studies

#### *In vitro* screen versus *Mtb* H_37_Ra and MAC NJ211

The initial screening set and selected other compounds were tested for their inhibitory activity against *Mtb* H_37_Ra (ATCC 25177) and MAC NJ211 strains. Screening was performed at 1.28 and 12.8 μg/mL in Middlebrook 7H9 broth supplemented with 0.2% glycerol and ADC enrichment using a colorimetric (Alamar blue) microdilution broth assay by reported methods.[15] Active compounds (≤12.8 μg/mL) were re-tested using twofold dilutions to obtain the actual MIC_99_. The MIC_99_ is defined as the lowest drug concentration that completely inhibited growth.

#### *In vitro* qHTS assay against *Mtb* H_37_Rv and cytotoxicity evaluations

The Sulindac set was screened against *Mtb* H_37_Rv to determine the IC_90_ (the concentration that inhibits bacterial growth by 90%) by reported methods.[36] These compounds were also counter screened at St. Jude Children’s Research Hospital for a comparative toxicity assessment against an established mammalian cell line relative to the *in vitro Mtb* H_37_Rv results. BJ, a normal human foreskin fibroblast cell line was purchased from the American Type Culture Collection, ATCC, Manassas, VA, USA and was cultured according to recommendations.[37] Cell culture medium was also purchased from ATCC. Cells were routinely tested for mycoplasma contamination using the MycoAlert Mycoplasma Detection Kit (Lonza). Approximately 1,000 BJ exponentially growing cells were plated per well (30 μL) in white polystyrene flat bottom sterile 384-well tissue culture treated plates (Corning, Tewksbury, MA, USA), and incubated overnight at 37°C in a humidified 5% CO_2_ incubator. Compound solutions (DMSO) were pin-transferred (V&P Scientific, San Diego, CA, USA) the following day. Cytotoxicity was determined following a 72-hr incubation of BJ cells with each compound at a concentration of 10 μM using Promega Cell Titer Glo Reagent according to the manufacturer’s recommendations. Luminescence was measured on an Envision plate reader (Perkin-Elmer).

#### Inhibition of *Mtb* FtsZ polymerization *in vitro*

Selected available compounds that were active *in vitro* against *Mtb* H_37_Rv were examined for their ability to inhibit the hypothetical target, *Mtb* FtsZ, and its mammalian homolog tubulin using methods described previously.[15] Compounds were initially screened at 100 μM in duplicate. Those that showed <20% activity at this concentration were deemed inactive. All other samples (≥ 20% inhibition) were screened in a dose response format to obtain IC_50_ values. Any compounds that were insoluble in the assay medium at ≥ 25 μM did not advance. Compounds considered active in the *Mtb* FtsZ assay were further screened in a tubulin polymerization assay as reported.[15]

#### *In vitro* Z-ring inhibition Studies: Bacterial strains and growth conditions

Construction of *M*. *tuberculosis* 41 (*Mtb* 41) FtsZ reporter strain was previously described.[38] It is a *Mtb* H_37_Ra derivative strain where the chromosomal *ftsZ* is deleted by homologous recombination and contains a copy of *Pami*::*ftsZ-gfp*. The *ftsZ-gfp* in this strain is expressed from the amidase promoter (*Pami*) and the reporter strain requires 0.2% acetamide for growth.[38] *Mtb* 41 was grown in Middlebrook 7H9 broth supplemented with oleic acid, albumin, dextrose, sodium chloride and 0.2% acetamide. In some experiments, actively growing cultures of *Mtb* 41 were exposed to the small-molecule inhibitors at 75 μM (ca. the H_37_Ra MIC_99_) and growth was followed for several days after exposure by monitoring the absorbance at 600 nm.

#### Microscopy

*M*. *tuberculosis* 41 strain was grown as described in the absence or presence of the Sulindac analogs for 72 hours, at indicated time points samples were harvested by centrifugation, washed in phosphate-buffered saline, fixed in 1% paraformaldehyde, and stored at 4°C until microscopy. Bacteria were examined by bright-field and fluorescence microscopy with a Nikon E600 microscope equipped with a 100X Nikon Plan Fluor oil immersion objective with a numerical aperture of 1.4 and a standard FITC filter set from Chroma. Images were acquired with a Photometrics Coolsnap ES camera and MetaMorph 6.2 imaging software (Universal Imaging Corporation). Images were optimized with Adobe Photoshop CS5.

### Computational methods

The *Mtb* FtsZ crystal structure (PDB code 1RLU) determined at 2.08 Å resolution was refined applying Prime preparation and refinement tools of the Protein preparation wizard as implemented in Schrödinger software. After the addition of hydrogens and detection of disulfide bonds the structure was optimized applying default parameters of the Impref utility using the OPLS2001 force field. The maximum allowed root-mean-square deviation between the refined structure and the input crystal structure was 0.3. Ligand structures were prepared using the LigPrep utility at pH 7.4. Ligands were docked into the interdomain cleft site using Induced Fit docking protocol in XP precision mode. Default parameters were applied except for setting the site enclosing box size to 33 Å. The Induced Fit docking method combines Glide docking with Prime structural refinement tools in the Schrödinger software to account for the flexibility of protein side chains within 5 Å of the ligand during docking.

## Results and Discussion

### A structurally diverse compound set selected for screening

**Fig 5:**
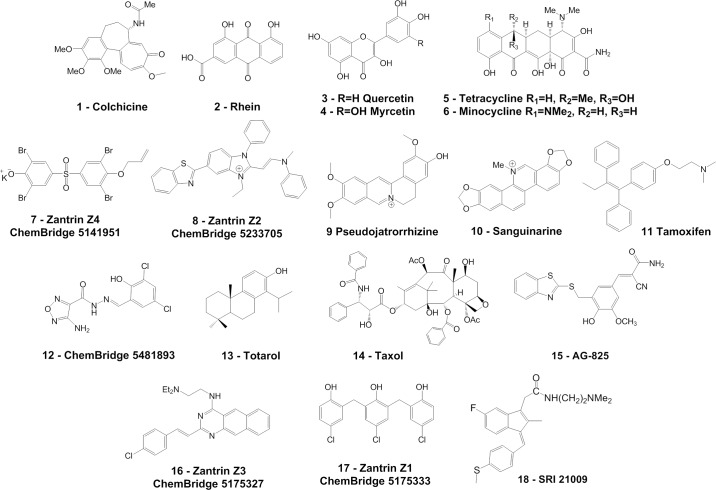
Structures of a diverse set of compounds selected for screening.

A diverse set of compounds and natural products was selected for screening against *Mtb* FtsZ and its mammalian homolog tubulin. In order to assess whole bacterial activity, MICs against the non-pathogenic mycobacterial strains *Mtb* H_37_Ra and MAC NJ211 were determined and inhibitory activities evaluated against a potential target, FtsZ, that is highly homologous between *Mtb* strains and essential in mycobacteria. Selected results and structures are given in Table 1 and Fig 5, respectively.

**Table 1:** *Mtb* FtsZ, tubulin and *Mtb* H_37_Ra data of selected compounds 1–18.

Entry	Name		*Mtb* FtsZ Polymerization IC_50_ (μM)^a^	Tubulin Polymerization IC_50_ (μM)^a^	*Mtb* H_37_Ra MIC_99_ (μg/mL)^b^	MAC NJ211 MIC_99_ (μg/mL)
**1**		Colchicine	104.4 ± 2.0	6.5 ± 0.9	>64	>64
**2**		Rhein	44.6 ± 6.3	NA	32 (112.66)^**b**^	>128
**3**		Quercetin dihydrate	21.7 ± 4.8	NA	>128	>128
**4**		Myrcetin	42.4 ± 6.3	NA	>128	>128
**5**		Tetracycline	87.1 ± 10.7	NA	1	ND
**6**		Minocycline	37.3 ± 4.6	NA	≤0.5	ND
**7**		Zantrin Z4	NA	77.0 ± 3.0	64	>64
**8**		Zantrin Z2	3.50 ± 0.3	NA	1 (2.05)^**b**^	1
**9**		Pseudojatrorrhizine	105.0 ± 32.0	NA	>64	64
**10**		Sanguinarine Cl‾ • H_2_O	23.0 ± 4.0	30.0 ± 7.0	64 (192.58)^**b**^	>64
**11**		Tamoxifen citrate	40.6 ± 17.9	NA	8 (21.53)^**b**^	32
**12**		ChemBridge 5481893	33.4 ± 3.0	NA	64 (202.47)^**b**^	>64
**13**		Totarol	47.5 ± 4.9	NA	25 (87.27)^**b**^	25
**14**		Taxol	NA	ND	16	ND
**15**		AG-825	31.7 ± 1.7	NA	ND	ND
**16**		Zantrin Z3	NA	NA	1	2
**17**		Zantrin Z1	NA	NA	8	16
**18**		SRI 21009	39.4 ± 4.9	NA	8 (19.49)^**b**^	16
STD*		Ethambutol	ND	ND	2–4	4–8

^a^Each compound was analyzed at least three times per assay and the mean ± the standard deviations are reported.

ND = not done.

NA = not active up to 100 μM.

^b^Concentration in μM provided in parentheses for selected actives.

*STD = Ethambutol antitubercular antibiotic standard.

Albendazole (not included above) is a vermicidal that binds to the colchicine site of parasite tubulin inhibiting polymerization into microtubules—NA in the FtsZ polymerization assay.

Colchicine was included as a known, albeit modest, inhibitor of *Mtb* FtsZ.[15] Rhein, Quercetin, Myrcetin, Tetracycline, and Minocycline (Aldrich Chemical Company) contain planar aromatic systems and bear resemblance to Viriditoxin,[39] a reported natural product that is active against *E*. *coli* FtsZ. Rhein and the isoflavones have also been reported to have antibacterial/antitubercular activity.[40] The Zantrins (Z1-5) were first reported by Margalit as inhibitors of bacterial FtsZ GTPase activity.[11] Z1-Z4 purchased from ChemBridge were included as a test to determine how well the reported GTPase activity correlates with inhibition of FtsZ polymerization as there is an apparent kinetic disconnect between GTPase effects and inhibition of polymerization.[41] In some respects, Zantrin Z2 is similar to the aromatic cationic compounds Sanguinarine and Pseudojatorrizine. Sanguinarine has been reported to show antibacterial activity and to inhibit *E*. *coli* FtsZ assembly.[42] Tamoxifen is a known antiestrogen drug that has effects on human tubulin and is reported to show antitubercular activity in a screen of the Prestwick collection of natural products and FDA approved drugs (see http://pubchem.ncbi.nlm.nih.gov).[43] Compound **12**, a 4-aminofurazan analog available from ChemBridge, is a reported inhibitor of *E*. *coli* FtsZ GTPase activity as well as protein polymerization in a standard light scattering assay.[44] Compound **12** is also reported to alter Z-ring assembly in whole bacterial cells and to cause bacterial filamentation in *E*. *coli* cells.[44] Totarol is a reported natural product that inhibits proliferation of a variety of bacteria and is reported to have activity against *Mtb* FtsZ *in vitro* as well as causing bacterial filamentation although recently reported as a promiscuous aggregator.[9,45] The taxanes are known drugs that alter tubulin polymerization dynamics, and analogs have been reported to show inhibition of *Mtb* growth *in vitro* and are reported to show specificity for *Mtb* FtsZ.[8] Compound AG-825 is a known inhibitor of HER-2 available from Calbiochem, and the compound bears striking resemblance to a reported class of bacterial FtsZ inhibitors out of the Stokes laboratory.[46]

### New analog series based on the Sulindac scaffold

We found that SSA (**18**), an early sulindac analog shows modest inhibition of *Mtb* FtsZ polymerization, inhibits bacterial growth but has no effect on human tubulin at 100 μM (Table 1). Further we explored *Mtb* FtsZ activities of several series of compounds designed based on the Sulindac scaffold (as described under Methods). The scope of these studies was to probe the effect of targeted modifications of this scaffold, the development of structure-activity relationships, and the identification of analogs with the most potent inhibition of FtsZ polymerization without affecting tubulin polymerization, coupled with potent whole cell antitubercular activity. Results of these studies are presented herein.

#### Whole cell antitubercular and cytotoxicity results

**Fig 6:**
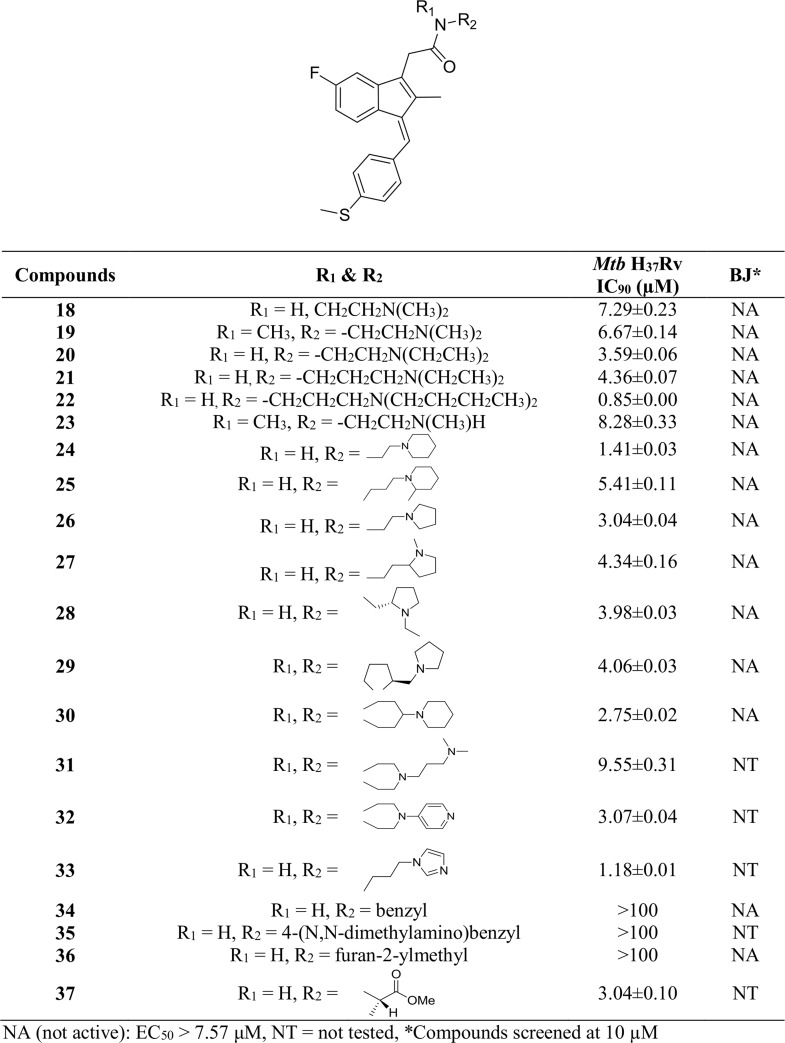
*Mtb* H_37_Rv and BJ cell data of Sulindac sulfide amide derivatives 18–37.

Fig 6 presents the *Mtb* H_37_Rv and BJ cell line cytotoxicity data of Sulindac sulfide amide derivatives **18–37**. Compounds **18–23** with basic acyclic acetamide linker at the C-3 position showed significant activity against the virulent strain H_37_Rv. Among these six compounds, compound **22** with two *n*-butyl groups at the terminal nitrogen was found to be more active than corresponding methyl and ethyl substituted compounds. It is notable from the screening data of compounds **20** and **21** that the carbon chain length between the acetamide and the terminal nitrogen has marginal influence on the activity. Either an additional substituent at the acetamide nitrogen or removal of a substituent from the terminal nitrogen did not improve the activity (**19** and **23**) dramatically. We also explored the antibacterial activity of compounds with a basic cyclic acetamide group at the C-3 position (**24–32**). The compounds showed no significant activity against the BJ mammalian cell line at the initial screening concentration similarly to results for the acyclic basic amide analogs **18–23**. Within this series, the piperidinylethyl analog **24** demonstrated the most significant *Mtb* whole cell activity. The imidazol-1-ylpropyl analog **33** showed potent activity against *Mtb* H_37_Rv. The compounds with aromatic (**34** and **35**) or heteroaromatic (**36**) groups at the acetamide linker are inactive in this assay. The activity of amino acid derivative **37** mirrored results for the acyclic and cyclic basic amides.

**Fig 7:**
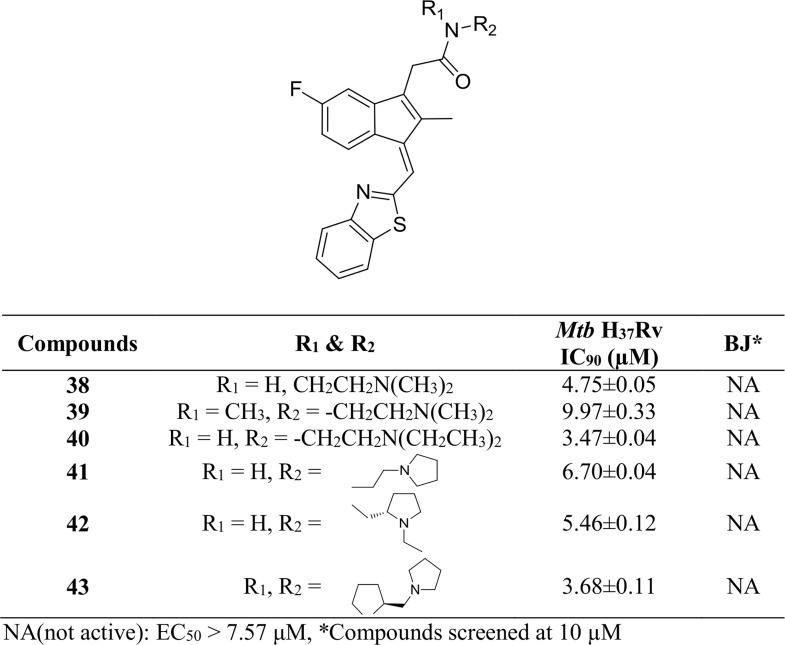
*Mtb* H_37_Rv and BJ cell data of benzothiazolyl Sulindac amide derivatives 38–43.

We then expanded our study to include a benzothiazol-2-ylmethylene group at the C-1 position (**38–43**, Fig 7) while varying the amide. These analogs demonstrated significant activity against *Mtb* H_37_Rv that was similar to their 4-methylthiobenzylidene analogs.

**Fig 8:**
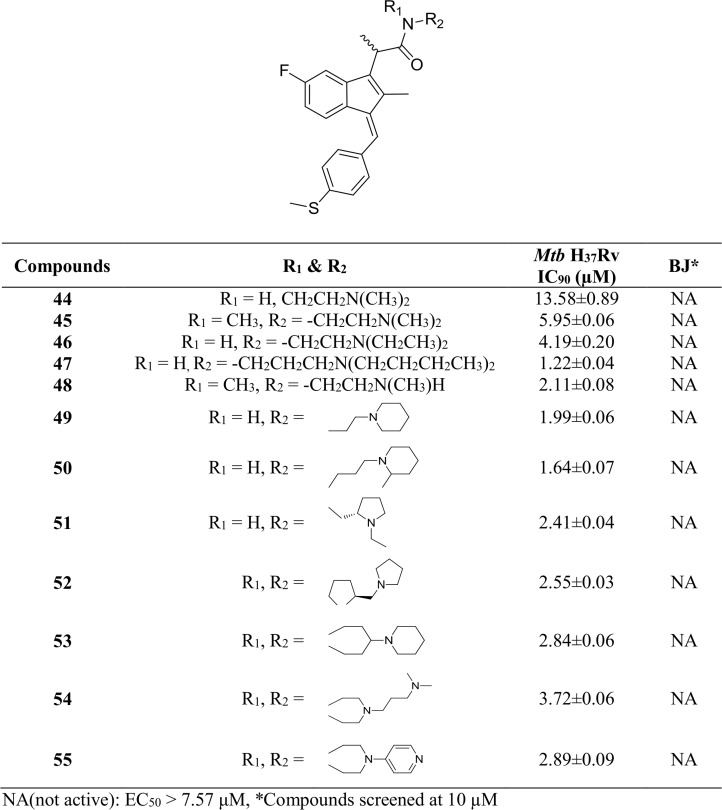
*Mtb* H_37_Rv and BJ cell data of α-methyl Sulindac sulfide amide derivatives 44–55.

Fig 8 lists the *Mtb* H_37_Rv and BJ cell data of α-methyl Sulindac sulfide amide derivatives **44–55**. Incorporation of a methyl group at the α-position of the amide group did not cause significant alterations in activity. The N,N-dimethylaminoethyl analog **44** was 2-fold less active than its desmethyl analog **18**. The N,N’- dimethylaminoethyl acetamide derivative **48**, in contrast, had 4-fold more activity than **23**. In the case of the cyclic basic amide series, the α-methyl analogs displayed similar or slightly better activity than their corresponding desmethyl analogs.

**Fig 9:**
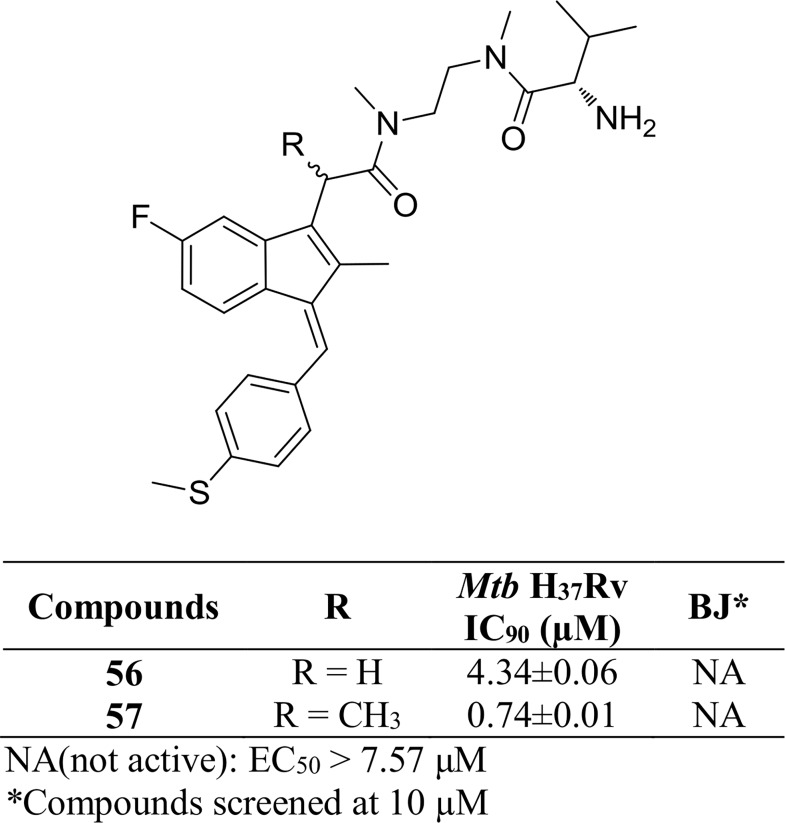
*Mtb* H_37_Rv and BJ cell data of extended Sulindac sulfide amide derivatives 56–57.

We also prepared a small set of extended Sulindac sulfide amide derivatives **56–57** from **23** and **48** by coupling with L-Valine. The α-methyl analog **57** displayed an IC_90_ of 0.74 μM against H_37_Rv, 6-fold more active than its desmethyl analog **56** (Fig 9).

**Fig 10:**
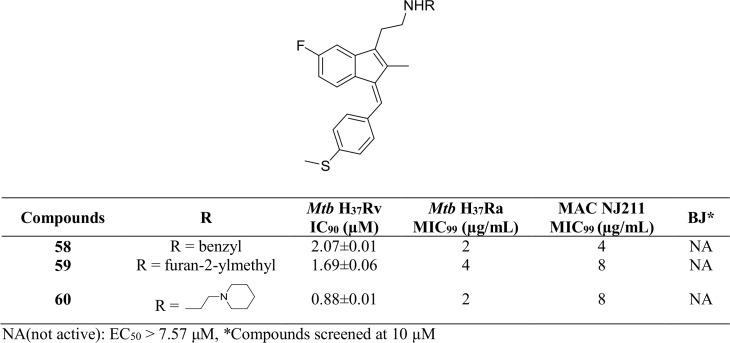
*Mtb* H_37_Rv and BJ cell data of Sulindac sulfide amine derivatives 58–60.

Analogs exploring the amine linkage at the C-3 position are presented in Fig 10. Compounds **58–59** with a benzyl or furan-2-ylmethyl linkage at the aminoethyl group at the C-3 position displayed excellent activity as compared to their inactive amide counterparts (**34** and **36**). The piperidinylethyl analog **60** also showed 1.6-fold more activity than its corresponding amide analog **24**.

**Fig 11:**
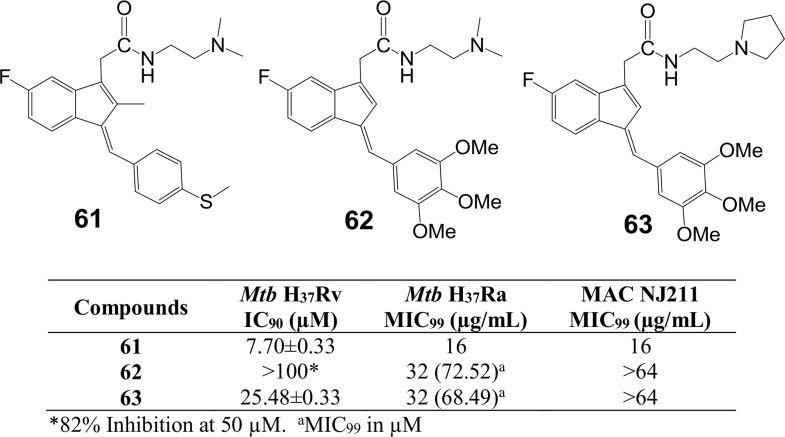
Whole cell activity of three E-conformers 61–63.

Finally, we prepared a small set of related E-conformation compounds **61–63**[31, 32] to further test activity against whole bacteria and *Mtb* FtsZ. These compounds and their activity are reported in Fig 11. Comparison of *Mtb* H_37_Rv activity between compounds **18** and **61** suggests no significant difference between the E and Z forms of this compound.

#### Activity of Sulindac Analogs against *Mtb* FtsZ and Tubulin

Selected compounds that were available in sufficient quantities were examined for their ability to inhibit both *Mtb* FtsZ and its mammalian homolog tubulin. The MIC for *Mtb* H_37_Ra was also determined for this set. Ten compounds demonstrated significant activity against *Mtb* FtsZ while not affecting tubulin polymerization and these are reported in Table 2. It is notable that the H_37_Ra and H_37_Rv (MIC_99_ versus IC_90_) data are relatively well correlated for these compounds.

**Table 2:** *Mtb* FtsZ, tubulin and *Mtb* H_37_Ra and Rv data of selected Sulindac derivatives.

Compounds	*Mtb* FtsZ Polymerization^a^ IC_50_ (μM)	Tubulin Polymerization^a^ IC_50_ (μM)	*Mtb* H_37_Rv IC_90_ (μM)	*Mtb* H_37_Ra MIC_99_ (μg/mL)
**18**	39.4 ± 4.9	NA	7.29±0.23	8
**19**	42.3 ± 6.0	NA	6.67±0.14	16
**23**	34.9 ± 7.0	NA	8.28±0.33	8
**24**	26.5 ± 4.8	NA	1.41±0.03	>4≤64
**26**	33.5 ± 5.9	NA	3.04±0.04	8
**35**	22.9 ± 4.8	NA	>100*	64
**56**	38.0 ± 6.3	NA	4.34±0.06	8
**61**	44.6 ± 6.3	NA	7.70±0.33	16
**62**	43.3 ± 9.8	NA	>100	32
**63**	37.6 ± 5.7	NA	25.48±0.33	32

^a^Each compound was analyzed at least three times per assay.

For the polymerization assays, the mean ± the standard deviations are reported.

NA = not active up to 100 μM.

*82% inhibition at 50 μM.

**Fig 12:**
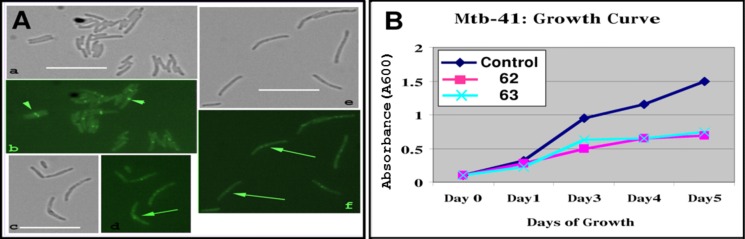
Compounds 62 and 63 inhibit FtsZ ring assembly and growth of *M*.*tuberculosis*. (A) Exponential cultures of *Mtb* 41 grown without **63** (a, b) or with **63** for 24 h (c, d) or 72 h (e, f) and imaged using brightfield (a, c, e) or fluorescent microscopy (b, d, f). Bar = 10 μms. Arrowhead–FtsZ-rings. Arrows–aberrant FtsZ structures. (B) Exponential cultures of *Mtb* 41 in 7H9 broth (-) or (+) inhibitors for 5 d. A_600_ measured on days listed and plotted.

To examine if the Sulindac analogs would also affect FtsZ polymerization *in vivo*, we utilized a *Mtb* FtsZ reporter strain, *Mtb* 41, wherein the FtsZ tagged with GFP serves as the sole source of FtsZ. In this strain the chromosomal *ftsZ* gene is deleted and a copy of *ftsZ-gfp* expressed from the inducible amidase promoter is integrated at the *attB* site.[38] Exponential cultures of *Mtb* 41 were exposed to **62** and **63** (75 μM) for 48 hours and the bacteria examined by brightfield and fluorescence microscopy (Fig 12 –data are given for **63**). Exposure to drug led to a clear increase in bacterial cell length (Fig 12, compare panel a to c). In addition, distinct midcell FtsZ-GFP rings seen in control cultures were absent in treated cultures (Fig 12, compare panel b with d). Instead, the compound treated bacteria showed weak FtsZ-GFP fluorescent spots or aggregates (Fig 12, panel d). Exposure to **62** and **63** also led to a similar growth inhibition of *M*. *tuberculosis* 41 (Fig 12B). Since FtsZ is essential for growth and viability of *Mtb*,[38] **62** and **63** inhibition of FtsZ ring assembly likely led to growth inhibition of *Mtb* 41.

#### Modeling *Mtb* FtsZ inhibitor binding

Sulindac sulfide is a known human COX-1/COX-2 inhibitor. Replacement of its carboxylate group with positively charged moieties such as N,N-dimethylamino ethyl amide in compound **18** results in analogs that have greatly reduced activity at COX-1/COX-2.[24] Tolerance and pharmacokinetic studies have shown that compound **18** shows a greatly improved toxicity profile compared to Sulindac sulfide.[24] While Sulindac sulfide is tolerated in mice up to 50 mg/kg over 20 days, compound **18** shows tolerance up to 300 mg/kg over 17 days. Also, compound **18** maintains ~4 μM plasma levels for 8 hours after gastric gavage at a dosage of 200 mg/kg.[24] Compounds presented in Table 2 are analogs of compound **18** containing the core substructure of Sulindac and positively charged amine moieties and therefore may be expected to have favorable pharmacokinetic and toxicity profiles as well. While *Mtb* FtsZ inhibitory activities of the presented Sulindac analogs are moderate, compounds from these series may serve as starting leads for the design of further chemical modifications to achieve better FtsZ inhibitory potencies. To provide insight into the possible binding site of the presented compounds we modeled representative analogs and known inhibitors from Table 1 into the crystal structure of *Mtb* FtsZ.

**Fig 13:**
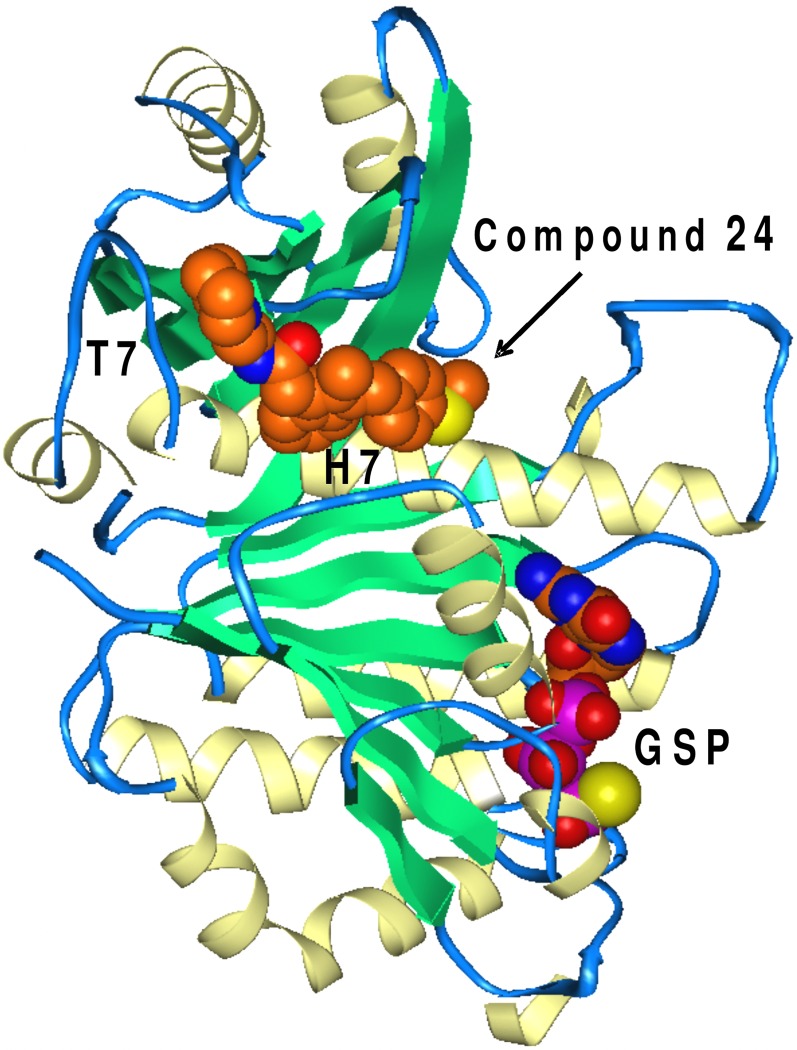
*Mtb* FtsZ crystal structure (PDB code 1RLU). Co-crystallized GSP in the GTP binding site and compound **24** docked into the interdomain cleft site are shown with carbons colored orange-brown.

Small molecule inhibitors may bind FtsZ in the GTP binding site or the interdomain cleft region (Fig 13), which is equivalent to the Taxol binding region in tubulin and also contains the co-crystallized PC190723 in *Staph*. *aureus* FtsZ (PDB code 4DXD). These two sites have been targeted in ligand binding studies of FtsZ inhibitor libraries.[47] Another pocket near loop T7 has also been investigated recently in case of a *B*. *subtilis* FtsZ inhibitor, BT-benzo-29.[48] Inhibitory effects of BT-benzo-29 on the assembly, GTPase activity of single mutant FtsZ constructs compared to wild type provided evidence that residues L272, V275 play an important role in the inhibition of FtsZ by this ligand. The corresponding residues in the *Mtb* FtsZ crystal structure (L269, I272, respectively) along with loop T7 define a new ligand binding site that is distinct from the interdomain cleft site. Further, docking studies suggested that the FtsZ inhibitors Scopoletin and CCR-11 may bind the same pocket.[49,50] In *Mtb* FtsZ the corresponding binding site is smaller compared to *B*. *subtilis* and we could not successfully dock Sulindac analogs into this site although we cannot exclude the possibility that Sulindac analogs may induce conformational changes of the T7 loop upon binding.

**Fig 14:**
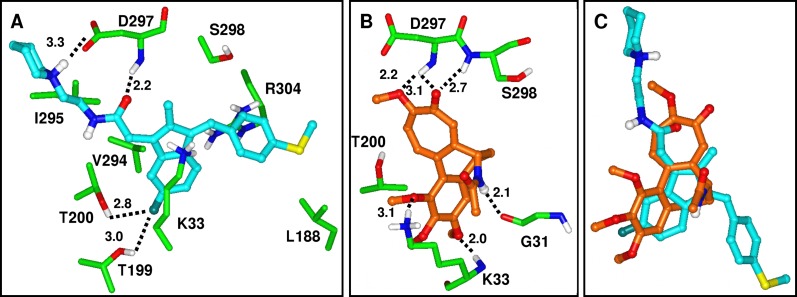
**Docked poses of (A) 24 (carbon atoms colored cyan) and (B) Colchicine (carbons colored orange).** All other atoms are colored by atom type (C green, O red, N blue, S yellow). H-bonds are indicated with dashed lines. (C) Docked poses of **24** and Colchicine displayed simultaneously show overlap of similar pharmacophoric features.

For docking Sulindac analogs we utilized the *Mtb* FtsZ crystal structure (PDB code 1RLU) and Induced Fit docking protocols (Schrödinger software), as detailed under Methods. Poses docked into the interdomain cleft region showed more favorable interactions and better Glide docking scores compared to poses docked into the GTP binding site. A representative analog (**24**) from Table 2 docked into the interdomain cleft is illustrated in Figs 13 and 14A. Compound **24** inhibits *Mtb* FtsZ polymerization with an IC_50_ of 26.5 μM and does not affect tubulin polymerization up to 100 μM. Compound **24** is favorably accommodated in the interdomain cleft binding site. The center of mass of the indene ring of compound **24** is at 4.0 Å distance from the amine of Lys33 indicating aromatic–charge stabilizing interactions. The fluorine substituent on the indene ring predicts polar interactions with Thr200 and Thr199. The terminal thiomethyl-benzene forms aromatic–positive charge contacts with Arg304 while the thiomethyl participates in non-polar interactions with Leu188. Replacement of the phenyl ring with a 4-pyridyl ring resulted in an inactive analog, consistent with the predicted loss of favorable interactions with Leu188. A 3,4,5-tri-OMe substitution introduced on the phenyl ring of compound **24** renders the corresponding analog inactive against *Mtb* FtsZ. The docked pose of **24** suggests that the 3,4,5-tri-OMe substitution could not be accommodated because the phenyl ring of **24** is ‘sandwiched’ between Arg304 and backbone atoms of Gln30, with Gln192 in close proximity. The piperidine ring of **24** participates in non-polar interactions with Ile295 and its amine moiety salt bridges to Asp297. Other analogs listed in Table 2 containing a variety of non-polar groups are accommodated in poses similar to that of **24**. The varying non-polar group in this series forms analogous, non-polar interactions with Ile295 while their positively charged amine is salt bridging with Asp297. The similarity of the binding poses and analogous non-polar interactions with Ile295 are consistent with our finding that these substitutions resulted in only a modest variation of *Mtb* FtsZ polymerization inhibition, as IC_50_s range between 22.9 and 44.6 μM within this series (Table 2).

Colchicine is a low potency inhibitor of *Mtb* FtsZ assembly at 104 μM and inhibits tubulin assembly at 6.5 μM.[15] While the Colchicine binding pocket of tubulin has no structurally analogous site in *Mtb* FtsZ, our docking results predict that Colchicine can also be accommodated in the interdomain cleft site of FtsZ. Similarities between the docked pose of compound **24** and Colchicine are illustrated in Fig 14B and 14C. Colchicine forms several favorable hydrogen bonding interactions with backbone atoms of Asp297, Ser298, Gly31, Lys33, and the sidechain of Lys33. Binding poses of Colchicine and compound **24** displayed simultaneously in Fig 14C suggest that the indene of compound **24** and the Colchicine core ring structure occupy overlapping space while the carbonyl and O-Me groups of the cyclohexyl moiety of Colchicine are in proximity to the amide carbonyl of analog **24**, both ligands participating in similar hydrogen bonding interactions with the D297 backbone.

Evidence supporting the interaction between inhibitors and residues in the interdomain cleft binding site region has been presented in the case of several inhibitors. Table 3 lists examples, showing implicated amino acids and the corresponding residues as mapped onto the *Mtb* FtsZ crystal structure (PDB code 1RLU). The *E*. *coli* FtsZ residues R33, D187 and E305 (corresponding *Mtb* G31, E185, E302, respectively) are involved in the binding of doxorubicin (Table 3). While G31 is in the interdomain cleft site, the other two *Mtb* FtsZ residues are located near the boundary of the interdomain cleft site region on the side opposite to loop T7. Ligands bound in the interdomain cleft side may possibly extend into this region near the *Mtb* residues E185, E302. Interestingly, a number of residues within the interdomain cleft binding site have been implicated in functional motions that affect FtsZ polymerization and activity.[51]

**Table 3:** FtsZ interdomain cleft site region residues implicated in inhibitor binding.

Species	Inhibitor	Implicated FtsZ residues	Corresponding *Mtb* residues	Reference
*Bacillus subtilis*	Plumbagin	D199, V307	D196, R304	[52]
*Bacillus cereus*	Cinnamaldehyde	V208, G295	V206, G292	[53]
*E*. *coli*	Doxorubicin	R33, D187, E305	G31, E185, E302	[54]

Out of the known FtsZ inhibitors from Table 1, we selected docked poses of five inhibitors that occupy approximately overlapping regions with Sulindac analogs while sharing common pharmacophoric features with the binding model of compound **24**: Colchicine (**1**), Zantrin Z2 (**8**), AG-825 (**15**), ChemBridge 5481893 (**12**), and Quercetin (**3**). Description of Induced Fit docked poses of the latter four inhibitors are provided under Supporting Information (S3 Appendix) and shown in the figure S1 Fig. Based on these five selected FtsZ inhibitors, a pharmacophore model was developed describing average positions of atom groups that form analogous interactions with *Mtb* FtsZ by inhibitors within this set. The obtained composite pharmacophore model may be utilized for the design of chemical modifications of the Sulindac scaffold (or other ligands) to mimic predicted interactions formed by this set of FtsZ inhibitors. A description and associated coordinates of the composite pharmacophore model are given in Supporting Information (S4 Appendix). The model is illustrated and tabulated in figure S2 Fig and S1 Table, respectively. The complete coordinates of the model are supplied in S4B Appendix.

## Conclusions

The series of compounds initially acquired and screened were selected based on established antibacterial activity and/or reported bacterial FtsZ inhibition, as well as similarity to other reported FtsZ inhibitors. All acquired samples were first screened in house against *Mtb* FtsZ and *Mtb* H_37_Ra and MAC NJ211, BSL-2 pathogens. Active samples were then tested against tubulin polymerization for selectivity determination. Table 1 presents results obtained for a number of compounds showing modest antitubercular activity versus *Mtb* H_37_Ra, coupled with activity against FtsZ in an IC_50_ range of about 20–50 μM. Results suggest further optimization strategies for some of these lead scaffolds that might improve FtsZ activity and potentially concurrent antibacterial activity against the bacillus. The Tetracycline (Tetracycline– **5** and Minocycline– **6**) class appears to show interesting activity against FtsZ, but the known primary target of this drug class is protein synthesis inhibition by binding the 30S ribosomal subunit, while the FtsZ activity is likely only an interesting sideline.

Among the remaining actives, three in particular stand out for different reasons. First, Zantrin Z2 (compound **8**) is approximately ten fold more potent against FtsZ than the range of activity of most other FtsZ actives in Table 1 (20–50 μM). Furthermore, it has the best antibacterial activity in the set with an MIC_99_ of 1 μM against *Mtb* H_37_Ra while showing no activity against tubulin up to 100 μM. Hence, we reasoned that initial results for this compound warranted further evaluation, and a sample was screened in a mammalian cell line toxicity assay (Vero cells) and a rapid PK model available through the NIH TAACF screening program. Unfortunately (see S1 Appendix and Table A in S1 Appendix, in Supporting Information for experimental details and results), Zantrin Z2 was equally toxic against Vero cells as it was active against *Mtb* H_37_Ra. Furthermore, at the dose of 300 mg/kg in a rapid PK mouse model, the sample showed no ill effects to the mice at a single dose but also showed no bioavailability by oral gavage, suggesting that it is likely a poor development candidate.

Two other compounds (**11** and **18**) stood out from the remainder of the active samples as purely synthetic molecules showing reasonable FtsZ activity and significant activity against *Mtb* H_37_Ra. Both compounds have advantages over the relatively more complex and less synthetically tractable natural product scaffolds that showed activity in Table 1. Additionally, Tamoxifen (**11**) is a clinically used agent suggesting reasonable starting bioavailability for a lead pharmacophore, and compound **18** is a simple Sulindac analog that has been reported as an active compound *in vivo* in a murine xenograft assay adapted for chemoprevention studies. It is interesting to note that several 1,1,2-triarylethenes akin to Tamoxifen show significant (nM) COX-2 activity,[55] demonstrating a plausible structural link of the NSAID Sulindac to Tamoxifen and a basis for their similar FtsZ inhibition as well as suggesting that certain of the related NSAIDs that effectively bind cyclooxygenases may have off-target FtsZ activity and possibly, depending on substitution, tubulin. In many respects the two scaffolds are similar in that they contain relatively planar aromatic ring systems with an attached basic amino function that could be protonated at physiological pH, suggesting that a positively charged group may be important for FtsZ activity. In fact, both of these features (aromaticity and cationic character) are common features within the screening actives, and are important parts of the phamacophore model developed in this program and discussed in this paper. While both samples appear to be reasonable starting points for further SAR, we chose to focus this work on the Sulindac scaffold due to our considerable experience with that template. In short, we prepared a small series of Sulindac derivatives designed to test several substitution points on the scaffold. New synthetic analogs were screened against *Mtb* H_37_Rv, and BJ cells (an immortalized human foreskin fibroblast cell line) for a preliminary assessment of overt cytotoxicity. Selected compounds were also tested against *Mtb* FtsZ, its mammalian homolog tubulin and *Mtb* H_37_Ra. Several observations can be made based on the modest SAR work. First, as a class, these compounds show good, lead activity against *Mtb* H_37_Rv *in vitro* with certain analogs showing IC_90_ values below 1.0 μM. Although these compounds showed no activity in the BJ cell toxicity assay at the modest screening cutoff, the Sulindac scaffold and analogs have been reported to have mammalian cell cytotoxicity albeit giving modest selectivity for cancer cell lines over normal cells *in vitro*.[24, 25] Finally, of the Sulindac analog set for which FtsZ activity was measured (Table 2), the H_37_Rv whole cell potency was consistently better than *Mtb* FtsZ inhibition activity in this series, which might be due to cell wall penetration/transport issues as well as possible activity at other *Mtb* targets. The difference between whole cell and FtsZ activities may be specific to the core scaffold shared within this series, since for example another scaffold, Zantrin Z2 shows about 10-fold more potent activity in the FtsZ assay than the average range of these analogs. Furthermore, the inhibition in this Sulindac series is in a modest IC_50_ range of approximately 23–45 μM in spite of the variety of substitutions, suggesting that we were not varying the structure enough to probe the true extent of the binding site pocket as demonstrated by retrospective docking results of representative analogs of this series.

In order to test that these samples actually had a true effect on FtsZ polymerization under normal bacterial growth conditions, we turned to the GFP-labeled Z-ring assay in Dr. Malini Rajagopalan’s laboratory. In a GFP labeled FtsZ reporter strain *Mtb* 41,[38] Sulindac analogs **62** and **63** showed significant growth inhibition at the MIC_99_ determined for those compounds in *Mtb* H_37_Ra. Furthermore, these growth effects translated into a clear visual inhibition of Z-ring formation on analysis of the fluorescently labeled protein aggregation and ring formation with time of exposure to both compounds. These results indicate that the Sulindac analogs **62** and **63** can have direct inhibitory effects on Z ring assembly, cell division and growth around the determined MIC_99_ in a related strain *Mtb* H_37_Ra.

Our docking results suggest that Sulindac analogs target an interdomain cleft site which is distinct from the GTP binding site in *Mtb* FtsZ. The presented model offers rational approaches to modify the scaffold to improve FtsZ binding through exploring this site more extensively while potentially improving *Mtb* versus host cell selectivity (e.g. against tubulin and off-target enzymes). Based on binding models of a structurally diverse set of known FtsZ inhibitors and their overlapping pharmacophoric features, we developed a composite pharmacophore model that describes shared interactions present in the same active site region. Another potential application is screening known *Mtb* phenotypic actives that are available on PubChem (http://www.ncbi.nlm.nih.gov/pcsubstance)[17,36] against the pharmacophore model in order to identify other potential FtsZ inhibitor scaffolds with known selective activity versus *Mtb* H_37_Rv. Coordinates of this pharmacophore model are available (S4 Appendix, Supporting Information) for future applications for the design of chemical modifications of inhibitor scaffolds for the improvement of their binding affinities. Overall, our observations indicate that Sulindac derivatives remain a promising development scaffold for the design of new FtsZ inhibitors against *M*. *tuberculosis*. Promising analogs in the various series presented are candidates for future medicinal chemistry optimization efforts and for further validation in follow-up assays.

## Supporting Information

S1 Appendix Follow-up Screening of Zantrin Z2. (DOCX)

S2 Appendix Synthetic experimental details. Additional details on experimental synthetic procedures.
(DOCX)

S3 Appendix Description of docked poses of a selected set of FtsZ inhibitors utilized for the development of the pharmacophore model. (DOCX)

S4 Appendix Pharmacophore model. (A) Description and (B) Cartesian coordinates.
(DOCX)

S1 Fig Docked poses of Zantrin Z2, Quercetin, AG-825, ChemBridge 5481893 (compound 12) at *Mtb* FtsZ. Carbons of the ligand are colored pink, all other atoms by atom type. H-bonds are illustrated with dashed lines.
(TIFF)

S2 Fig Pharmacophore model developed based on five selected *Mtb* FtsZ inhibitors. (A) Pharmacophore model sites are shown. Interactions with FtsZ residues are indicated with dashed lines. Hydrogen bond donor/acceptor D/A sites are shown as orange colored spheres, acceptor A site in magenta and aromatic site in cyan. (B) Positions of the docked poses of compounds 12 and 24 in relation to pharmacophoric sites are shown.
(TIFF)

S1 Table Pharmacophore sites 1–10. Listed are: type of interactions, *Mtb* FtsZ residues involved and compounds contributing to each site. Residues interacting through backbone atoms only are marked with (b).
(DOCX)
