# Supplementary material for: Screening and Development of New Inhibitors of FtsZ from M. Tuberculosis
Source: PLoS One. 2016 Oct 21;11(10):e0164100. doi: 10.1371/journal.pone.0164100 (PMC5074515; doi:10.1371/journal.pone.0164100)
Supplement: S1 Appendix — (DOCX) [file pone.0164100.s001.docx]

**Supporting Information:**

**S1 Appendix. Follow-up Screening of Zantrin Z2**

**Screening and development of New Inhibitors of FtsZ from *M. tuberculosis***

Bini Mathew,^3^ Judith Varady Hobrath,^4^ Larry Ross,^3^ Michele C. Connelly,^5^ Hava Lofton,^6, 7^ Malini Rajagopalan,^6^ R. Kiplin Guy,^5^ and Robert C. Reynolds^1,2*^

^1^Department of Chemistry, The University of Alabama at Birmingham, Birmingham, Alabama 35294, USA

^2^Division of Hematology and Oncology, The University of Alabama at Birmingham, Birmingham, Alabama 35294, USA

^3^Drug Discovery Division, Southern Research Institute, 2000 Ninth Avenue South, Birmingham, AL 35205, USA

^4^Drug Discovery Unit, College of Life Sciences, University of Dundee, Dundee DD1 5EH, United Kingdom

^5^Dept. Chemical Biology & Therapeutics, St Jude Children's Research Hospital, 262 Danny Thomas Place, Memphis, TN 38105, USA

^6^The University of Texas Health Science Center at Tyler, Tyler, Texas 75708, USA

^7^Current address: Department of Medical Biochemistry and Microbiology, Uppsala University, SE-75123 Uppsala Sweden

**Studies evaluating Zantrin Z2 as a potential antibacterial scaffold**

Zantrin Z2 was reported as a bacterial FtsZ GTPase inhibitor [see reference 11 in full paper]. We selected this compound for further studies considering its potent effect on FtsZ polymerization coupled with potent *Mtb* H37Ra growth inhibition and no activity against tubulin polymerization up to 100 µM (full paper - Table 1).

**Zantrin Z2: Cytotoxicity against Vero cells**

In order to progress Zantrin Z2 into advanced animal assays through the NIH Tuberculosis Antimicrobial Acquisition and Coordinating Facility (TAACF) it was necessary to first screen this sample in activity and cytotoxicity assays through the NIH TAACF program. Hence, concurrent with the determination of an IC_90_, Zantrin Z2 was tested by the TAACF for cytotoxicity (CC_50_) in Vero cells using Promega non-radioactive cell proliferation assay kits by reported methods.[ see reference 36 in full paper] The comparable toxicity of Zantrin Z2 in *Mtb* H_37_Rv and Vero cell assays raises concern for the general cytotoxicity of this compound (Table A in S1 Appendix), reflected also in its low selectivity index (SI) value, which is defined as the ratio of the measured CC_50_ in Vero cells to the IC_90_ (against *Mtb* H_37_Rv).

Table A in S1 Appendix. Advanced assay data for Zantrin Z2 is shown below.

| In vitro whole cell data | | | | PK Data | | |
| --- | --- | --- | --- | --- | --- | --- |
| Alamar blue Assay | *Mtb* H_37_Rv IC_90_ (µg/mL) | Vero cell toxicity CC_50_ (µg/mL) | Selectivity Index (CC_50_/IC_90_) | Route | Dose (mg/kg) | Bioavailability |
|  | 1.56 (3.20)* | 1.70 (3.49)* | 1.09 | Oral gavage | 300 | None |

*Concentration in µM in parentheses

**Zantrin Z2: *In vivo* PK studies**

In spite of the poor selectivity in the Vero cell cytotoxicity assay, this compound was advanced *in vivo* to determine toxicity and bioavailability in mice prior to efficacy screening against a murine *Mtb* model in order to evaluate this scaffold as further candidate for antitubercular drug discovery. Hence, Zantrin Z2 was initially evaluated for *in* *vivo* toxicity using an acute toxicity mouse model at 100, 300 and 500 mg/kg. At 300 mg/kg, the lowest dose not showing behavioral effects or lethality, Zantrin Z2 gave no bioavailability. The data for Zantrin Z2 as provided through the TAACF is summarized in Table A in S1 Appendix.

Our results suggest that while Zantrin Z2 can be useful as a probe of *Mtb* FtsZ inhibition, it is unlikely, however, to serve as a suitable lead for future development.
