# Supplementary material for: Screening and Development of New Inhibitors of FtsZ from M. Tuberculosis
Source: PLoS One. 2016 Oct 21;11(10):e0164100. doi: 10.1371/journal.pone.0164100 (PMC5074515; doi:10.1371/journal.pone.0164100)
Supplement: S2 Appendix — Additional details on experimental synthetic procedures. (DOCX) [file pone.0164100.s002.docx]

**Supporting Information:**

**S2 Appendix. Synthetic experimental details.**

**Screening and development of New Inhibitors of FtsZ from *M. tuberculosis***

Bini Mathew,^3^ Judith Varady Hobrath,^4^ Larry Ross,^3^ Michele C. Connelly,^5^ Hava Lofton,^6, 7^ Malini Rajagopalan,^6^ R. Kiplin Guy,^5^ and Robert C. Reynolds^1,2*^

^1^Department of Chemistry, The University of Alabama at Birmingham, Birmingham, Alabama 35294, USA

^2^Division of Hematology and Oncology, The University of Alabama at Birmingham, Birmingham, Alabama 35294, USA

^3^Drug Discovery Division, Southern Research Institute, 2000 Ninth Avenue South, Birmingham, AL 35205, USA

^4^Drug Discovery Unit, College of Life Sciences, University of Dundee, Dundee DD1 5EH, United Kingdom

^5^Dept. Chemical Biology & Therapeutics, St Jude Children's Research Hospital, 262 Danny Thomas Place, Memphis, TN 38105, USA

^6^The University of Texas Health Science Center at Tyler, Tyler, Texas 75708, USA

^7^Current address: Department of Medical Biochemistry and Microbiology, Uppsala University, SE-75123 Uppsala Sweden

**Synthetic experimental details.**

Anhydrous solvents and reagents from Aldrich were used without further drying. Reactions were monitored by thin-layer chromatography (TLC) on precoated E. Merck silica gel (60F254) plates (0.25 mm) and visualized using UV light (254 nm). Flash chromatography was carried out on Fischer silica gel G 60 (230-400 mesh). Purification of certain compounds was carried out by utilizing a Teledyne Isco Combiflash® Rf automated chromatography machine. Melting points, determined with an OptiMelt Automated Melting Point System and, are uncorrected. The exact mass spectral data were obtained with an Agilent LC-MSTOF or with Bruker BIOTOF II by electrospray ionization (ESI). ^1^HNMR spectra were recorded on a Nicolet NT-300 NB spectrometer operating at 300.635 MHz or on Agilent/Varian MR-400 spectrometer operating at 399.930 MHz. Chemical shifts in CDCl_3_ and Me_2_SO-d_6_ are expressed in parts per million downfield from tetramethylsilane (TMS) Chemical shifts (δ) listed for multiplets were measured from the approximate centers, and relative integrals of peak areas agreed with those expected for the assigned structures. Determination of % purity were obtained by HPLC using an Agilent 1100 LC equipped with a diode array UV detector and monitored at multiple wavelengths. Microanalyses were performed on a Perkin-Elmer 2400 CHN analyzer. ESI-MS spectra were recorded on a BioTof-2 time-of-flight mass spectrometer.

**Method 1**

O-(Benzotriazol-1-yl)-N,N,N’,N’-tetramethyluronium hexafluoro phosphate, (HBTU) (1.2 equivalents) was added to a solution of sulindac (1 equivalent), the appropriate amine (1.5 equivalents) and Et_3_N (2 equivalents) in dry acetonitrile (10 mL) at room temperature under argon atmosphere. The reaction mixture was stirred at room temperature for 1-2 h. Solvent was removed under reduced pressure and the crude product was purified by flash column chromatography (60-200 mesh) to afford the amide in excellent yield.

**Method 2**

(2-(7-Aza-1H-benzotriazole-1-yl)-1,1,3,3-tetramethyluronium hexafluorophosphate), (HATU) (1.2 equivalents) was added to a solution of acid (1 equivalent), the appropriate amine (1.5 equivalents) and DIEA (2 equivalents) in dry acetonitrile (10 mL) at room temperature under argon atmosphere. The reaction mixture was stirred at room temperature for 1-2 h. Solvent was evaporated under reduced pressure and the crude product was purified using a Teledyne Isco Combiflash® Rf purification machine to provide the desired amide in excellent yield.

**Method 3**

To a suspension of acid (1 equivalent) in dry methanol at 0 ^o^C was slowly added thionyl chloride (1.5 equivalents). The reaction mixture was stirred at room temperature for 3h. Methanol was removed and the crude product was then washed with aqueous NaHCO_3_, and the solution and extracted with CHCl_3_ (2 x 25 mL). The combined organic extracts were dried over anhydrous Na_2_SO_4_ and the solvent was evaporated under reduced pressure. Pure product was separated by crystallization (90-96% yield).

**Method 4**

LDA (1.5 equivalents) was added to a precooled solution of sulindac methyl ester in dry THF at -78 ^o^C under argon atmosphere. The enolate solution was stirred for 10 minutes under the same conditions and methyl iodide (1.3 equivalents) was added slowly to the reaction mixture. The mixture was stirred for 3 h at -78 ^o^C and then allowed to warm to room temperature overnight. THF was evaporated and the residue portioned between saturated aqueous NH_4_Cl solution and CH_2_Cl_2_. The aqueous layer was extracted twice with CH_2_Cl_2_, the combined CH_2_Cl_2_ extracts were dried over anhydrous Na_2_SO_4_, and solvent was evaporated to give the crude product. Purification was carried out using a Teledyne Isco Combiflash® Rf purification machine to provide α-methylsulindac ester in excellent yield (73-93% yield).

**Method 5**

KOH (3 equivalents) in H_2_O (20 mL) was added to a solution of α-methylsulindac ester in ethanol (20 mL) and stirred at room temperature for 12 hours. The reaction mixture was washed with 2N HCl to neutralize excess base and was then extracted with CHCl_3_ (3 x 25 mL). The combined organic extracts were dried over anhydrous Na_2_SO_4_ and the solvent was evaporated under reduced pressure. The crude product was purified using a Teledyne Isco Combiflash® Rf purification machine to afford α-methylsulindac in quantitative yield.

**Method 6**

To a solution of ester (1 equivalent) in dry toluene at -70 ^o^C under argon atmosphere was slowly added diisobutyl aluminum hydride (1 M) in toluene (1.2 equivalents) and the resulting mixture was stirred at -70 ^o^C for 1-2 hours. Methanol (10 mL) was added slowly at -70 ^o^C and the solution was allowed to warm to room temperature. The reaction mixture was washed with 1N aqueous HCl and extracted with CH_2_Cl_2_ (2 x 20 mL). The combined organic fractions were dried over anhydrous Na_2_SO_4_ and evaporated in *vacuuo*. The crude aldehyde was used for the next step without further purification.

**Method 7**

Aldehyde (1 equivalent) and amine (1.5 equivalents) were mixed in dry MeOH at room temperature under argon atmosphere. The reaction mixture was stirred at room temperature and progress of the reaction was monitored by TLC. After the complete formation of aldimine (3-5 h), NaBH_4_ (1.5 equivalents) was added slowly at room temperature. The reaction mixture was stirred for 15 more minutes and quenched with 1N NaOH. The product was extracted with CH_2_Cl_2_ (3 x 20 mL) and dried over anhydrous Na_2_SO_4_. The solvent was evaporated in *vacuuo* and purified by column chromatography to afford sulindac amine as yellow viscous liquid.

**(Z)-N-(2-(dimethylamino)ethyl)-2-(5-fluoro-2-methyl-1-(4-(methylthio)benzylidene)-1H-inden-3-yl)acetamide (18)**

By following method 1, the title compound **18** was obtained as a yellow crystalline solid. mp 138-140 ^o^C. ESI-MS m/z: 411.20 [M+H]^+^. ^1^H NMR (CDCl_3_, 300 MHz): δ 7.45 (2H, d, J = 8.1 Hz, 3’-H, 5’-H), 7.39 (1H, dd, J = 5.1 Hz, 8.4 Hz, 7-H), 7.30 (2H, d, J = 8.4 Hz, 2’-H, 6’-H), 7.15 (1H, s, 10-H), 6.86 (1H, dd, J = 2.4 Hz, 9.0 Hz, 4-H), 6.60 (1H, td, J = 2.7 Hz, 9.3 Hz, 6-H), 6.17 (1H, bs, NH), 3.50 (2H, s, 3-CH_2_), 3.27 (2H, q, J = 6.0 Hz, CO-NHCH_2_CH_2_), 2.55 (3H, s, SCH_3_), 2.31 (2H, t, J = 6.3 Hz, CO-NH-CH_2_-CH_2_), 2.20 (3H, s, 2-CH_3_), 2.10 (3H, s, -N(CH_3_)_2_). Anal. calcd for [C_24_H_27_FN_2_OS]: C, 70.21; H, 6.63; N, 6.82. Found: C, 69.84; H, 6.77; N, 6.89.

**(Z)-N-(2-(Dimethylamino)ethyl)-2-(5-fluoro-2-methyl-1-(4-(methylthio)benzylidene)-1H-inden-3-yl)-N-methylacetamide (19)**

By following method 1, the title compound **19** was obtained as a yellow viscous liquid in 98% yield. ESI-MS m/z: 425.23 [M+H]^+^. ^1^H NMR (CDCl_3_, 300 MHz): δ 7.43 (2H, d, J = 8.1 Hz, 3’-H, 5’-H), 7.33 (1H, dd, J = 5.4 Hz, 8.4 Hz, 7-H), 7.28 (2H, d, J = 8.1 Hz, 2’-H, 6’-H), 7.09 (1H, s, 10-H), 6.91 (1H, dd, J = 2.1 Hz, 9.0 Hz, 4-H), 6.55 (1H, td, J = 2.4 Hz, 9.0 Hz, 6-H), 3.59 (2H, s, 3-CH_2_), 3.51 (2H, t, J = 6.9 Hz, CO-N(CH_3_)CH_2_CH_2_), 3.09 (3H, s, CO-N(CH_3_)CH_2_), 2.54 (3H, s, SCH_3_), 2.44 (2H, t, J = 6.9 Hz, CO-N(CH_3_)-CH_2_-CH_2_), 2.25 (3H, s, -N(CH_3_)_2_), 2.24 (3H, s, -N(CH_3_)_2_), 2.17 (3H, s, 2-CH_3_). Anal. calcd for [C_25_H_29_FN_2_OS]: C, 70.72; H, 6.88; N, 6.60. Found: C, 70.80; H, 7.10; N, 6.41.

**(Z)-N-(2-(Diethylamino)ethyl)-2-(5-fluoro-2-methyl-1-(4-(methylthio)benzylidene)-1H-inden-3-yl)acetamide (20)**

By following method 1, the title compound **20** was obtained as a yellow solid in 97% (LCMS purity: 100%) yield. mp 98-100 ^o^C. ESI-MS m/z: 439.25 [M+H]^+^. ^1^H NMR (CDCl_3_, 300 MHz): δ 7.42 (2H, d, J = 8.1 Hz, 2’-H, 6’-H), 7.38 (1H, dd, J = 5.1 Hz, 8.4 Hz, 7-H), 7.30 (2H, d, J = 8.4 Hz, 3’-H, 5’-H), 7.15 (1H, s, 10-H), 6.85 (1H, dd, J = 2.4 Hz, 9.0 Hz, 4-H), 6.59 (1H, td, J = 2.4 Hz, 9.0 Hz, 6-H), 6.49 (1H, bs, NH), 3.50 (2H, s, 3-CH_2_), 3.22 (2H, q, J = 5.4 Hz, CONH-CH_2_), 2.55 (3H, s, SCH_3_), 2.42 (2H, t, J = 6.0 Hz, CH_2_-CH_2_N), 2.33 (4H, q, J = 6.9 Hz, -N(CH_2_-CH_3_)_2_), 2.19 (3H, s, 2-CH_3_), 0.77 (6H, t, J = 7.2 Hz, -N(CH_2_-CH_3_)_2_). HRMS calcd for [C_26_H_31_FN_2_OS+H]^+^: 439.22139, Found: 439.22167.

**(Z)-N-(3-(Diethylamino)propyl)-2-(5-fluoro-2-methyl-1-(4-(methylthio)benzylidene)-1H-inden-3-yl)acetamide (21)**

By following method 1, the title compound **21** was obtained as a yellow solid in 98% (LCMS purity: 100%) yield. mp 78-81 ^o^C. ESI-MS m/z: 453.25 [M+H]^+^. ^1^H NMR (CDCl_3_, 300 MHz): δ 7.43 (2H, d, J = 8.1 Hz, 2’-H, 6’-H), 7.38 (1H, dd, J = 5.1 Hz, 8.4 Hz, 7-H), 7.30 (2H, d, J = 8.7 Hz, 3’-H, 5’-H), 7.14 (1H, s, 10-H), 7.13 (1H, bs, NH), 6.86 (1H, dd, J = 2.4 Hz, 8.7 Hz, 4-H), 6.59 (1H, td, J = 2.4 Hz, 9.0 Hz, 6-H), 3.49 (2H, s, 3-CH_2_), 3.32 (2H, q, J = 5.4 Hz, CONH-CH_2_), 2.55 (3H, s, SCH_3_), 2.38 (2H, t, J = 6.0 Hz, CH_2_-CH_2_N), 2.31 (4H, q, J = 7.2 Hz, -N(CH_2_-CH_3_)_2_), 2.19 (3H, s, 2-CH_3_), 1.60-1.52 (2H, m, CH_2_-CH_2_-N(Et)_2_), 0.83 (6H, t, J = 7.2 Hz, -N(CH_2_-CH_3_)_2_). HRMS calcd for [C_27_H_33_FN_2_OS+H]^+^: 453.23704, Found: 453.23762.

**(Z)-N-(3-(Dibutylamino)propyl)-2-(5-fluoro-2-methyl-1-(4-(methylthio)benzylidene)-1H-inden-3-yl)acetamide (22)**

By following method 1, the title compound **22** was obtained as a yellow solid in 92% yield. mp 81-83 ^o^C. ESI-MS m/z: 509.30 [M+H]^+^. ^1^H NMR (CDCl_3_, 300 MHz): δ 7.44 (2H, d, J = 8.1 Hz, 2’-H, 6’-H), 7.39 (1H, dd, J = 5.1 Hz, 8.4 Hz, 7-H), 7.29 (2H, d, J = 8.4 Hz, 3’-H, 5’-H), 7.18 (1H, bs, NH), 7.14 (1H, s, 10-H), 6.85 (1H, dd, J = 2.4 Hz, 9.0 Hz, 4-H), 6.59 (1H, td, J = 2.4 Hz, 9.3 Hz, 6-H), 3.49 (2H, s, 3-CH_2_), 3.32 (2H, q, J = 5.7 Hz, CONHCH_2_), 2.55 (3H, s, SCH_3_), 2.38 (2H, t, J = 6.0 Hz, CH_2_N(CH_2_-CH_2_-CH_2_-CH_3_)_2_), 2.20 (4H, t, J = 7.5 Hz, CH_2_N(CH_2_-CH_2_-CH_2_-CH_3_)_2_), 2.19 (3H, s, 2-CH_3_), 1.58-1.50 (2H, m, CH_2_CH_2_NBu_2_), 1.24-1.10 (8H, m, N(CH_2_-CH_2_-CH_2_-CH_3_)_2_), 0.85 (6H, t, J = 6.9 Hz, N(CH_2_-CH_2_-CH_2_-CH_3_)_2_). Anal. calcd for [C_31_H_41_FN_2_OS]: C, 73.19; H, 8.12; N, 5.51. Found: C, 72.81; H, 8.34; N, 5.28.

**(Z)-2-(5-Fluoro-2-methyl-1-(4-(methylthio)benzylidene)-1H-inden-3-yl)-N-methyl-N-(2-(methylamino)ethyl)acetamide (23)**

By following method 1, the title compound **23** was obtained as a yellow viscous liquid in 82% (LCMS purity: 97.4%) yield. ESI-MS m/z: 411.26 [M+H]^+^, 821.49 [2M+H]^+^. ^1^H NMR (CDCl_3_, 300 MHz): δ 7.43 (2H, d, J = 8.1 Hz, 3’-H, 5’-H), 7.33 (1H, dd, J = 5.4 Hz, 8.4 Hz, 7-H), 7.28 (2H, d, J = 8.4 Hz, 2’-H, 6’-H), 7.10 (1H, s, 10-H), 6.93 (1H, dd, J = 2.1 Hz, 9.3 Hz, 4-H), 6.56 (1H, td, J = 2.4 Hz, 9.0 Hz, 6-H), 3.69 (2H, s, 3-CH_2_), 3.53 (2H, t, J = 6.3 Hz, CO-N(CH_3_)-CH_2_-CH_2_), 3.09 (3H, s, CO-N(CH_3_)-), 2.76 (2H, t, J = 6.3 Hz, CO-N(CH_3_)-CH_2_-CH_2_), 2.54 (3H, s, SCH_3_), 2.45 (3H, s, -NH(CH_3_)), 2.18 (3H, s, 2-CH_3_). HRMS calcd for [C_24_H_27_FN_2_OS+H]^+^: 411.19009, Found: 411.18995.

**(Z)-2-(5-Fluoro-2-methyl-1-(4-(methylthio)benzylidene)-1H-inden-3-yl)-N-(2-(piperidin-1-yl)ethyl)acetamide (24)**

By following method 1, the title compound **24** was obtained as a yellow solid in 97% (LCMS purity: 99.5%) yield. mp 130-133 ^o^C. ESI-MS m/z: 451.30 [M+H]^+^. ^1^H NMR (CDCl_3_, 300 MHz): δ 7.42 (2H, d, J = 8.1 Hz, 2’-H, 6’-H), 7.39 (1H, dd, J = 5.1 Hz, 8.4 Hz, 7-H), 7.31 (2H, d, J = 8.4 Hz, 3’-H, 5’-H), 7.17 (1H, s, 10-H), 6.85 (1H, dd, J = 2.4 Hz, 8.7 Hz, 4-H), 6.60 (1H, td, J = 2.4 Hz, 9.0 Hz, 6-H), 6.48 (1H, s, NH), 3.51 (2H, s, 3-CH_2_), 3.24 (2H, q, J = 5.7 Hz, NH-CH_2_-CH_2_), 2.55 (3H, s, SCH_3_), 2.29 (2H, t, J = 6.0 Hz, NH-CH_2_-CH_2_), 2.21 (3H, s, 2-CH_3_), 2.17 (4H, bs, 2’’-H, 6’’-H), 1.28 (6H, bs, 3’’-H, 4’’-H, 5’’-H). HRMS calcd for [C_27_H_31_FN_2_OS+H]^+^: 451.22139, Found: 451.22055.

**(Z)-2-(5-Fluoro-2-methyl-1-(4-(methylthio)benzylidene)-1H-inden-3-yl)-N-(3-(2-methylpiperidin-1-yl)propyl)acetamide (25)**

By following method 1, the title compound **25** was obtained as a yellow solid in 96% yield. mp 84-86 ^o^C. ESI-MS m/z: 479.26 [M+H]^+^. ^1^H NMR (CDCl_3_, 300 MHz): δ 7.43 (2H, d, J = 8.1 Hz, 2’-H, 6’-H), 7.37 (1H, dd, J = 5.4 Hz, 8.7 Hz, 7-H), 7.28 (2H, d, J = 8.4 Hz, 3’-H, 5’-H), 7.17 (1H, s, 10-H), 7.00 (1H, bs, NH), 6.90 (1H, dd, J = 2.4 Hz, 8.7 Hz, 4-H), 6.57 (1H, td, J = 2.4 Hz, 9.0 Hz, 6-H), 3.54 (2H, s, 3-CH_2_), 3.34-2.67 (7H, m, NH-CH_2_-CH_2_-CH_2_, 2’’-H, 6’’-H), 2.54 (3H, s, SCH_3_), 2.22 (3H, s, 2-CH_3_), 2.03-1.49 (8H, m, NH-CH_2_-CH_2_, 3’’-H, 4’’-H, 5’’-H), 1.19 (3H, d, J = 6.6 Hz, 2’’-CH_3_). HRMS calcd for [C_29_H_35_FN_2_OS+H]^+^: 479.25267, Found: 479.25318.

**(Z)-2-(5-Fluoro-2-methyl-1-(4-(methylthio)benzylidene)-1H-inden-3-yl)-N-(2-(pyrrolidin-1-yl)ethyl)acetamide (26)**

By following method 1, the title compound **26** was obtained as a yellow solid in 98% (LCMS purity: 99.8%) yield. mp 106-108 ^o^C. ESI-MS m/z: 437.23 [M+H]^+^. ^1^H NMR (CDCl_3_, 300 MHz): δ 7.44 (2H, d, J = 8.1 Hz, 3’-H, 5’-H), 7.39 (1H, dd, J = 5.4 Hz, 8.7 Hz, 7-H), 7.30 (2H, d, J = 8.4 Hz, 2’-H, 6’-H), 7.15 (1H, s, 10-H), 6.85 (1H, dd, J = 2.4 Hz, 9.0 Hz, 4-H), 6.60 (1H, td, J = 2.4 Hz, 9.0 Hz, 6-H), 6.20 (1H, s, NH), 3.51 (2H, s, 3-CH_2_), 3.28 (2H, q, J = 5.7 Hz, NH-CH_2_-CH_2_), 2.55 (3H, s, SCH_3_), 2.49 (2H, t, J = 6.3 Hz, NH-CH_2_-CH_2_), 2.37-2.31 (4H, m, 2’’-H, 5’’-H), 2.19 (3H, s, 2-CH_3_), 1.63-1.57 (4H, m, 3’’-H, 4’’-H). HRMS calcd for [C_26_H_29_FN_2_OS+H]^+^: 437.20574, Found: 437.20572.C_29_H_35_FN_2_OS+H]^+^: 479.25267, Found: 479.25318.

**(Z)-2-(5-Fluoro-2-methyl-1-(4-(methylthio)benzylidene)-1H-inden-3-yl)-N-(2-(1-methylpyrrolidin-2-yl)ethyl)acetamide (27)**

By following method 1, the title compound **27** was obtained as a yellow viscous liquid in 97% (LCMS purity: 100%) yield. ESI-MS m/z: 451.26 [M+H]^+^. ^1^H NMR (CDCl_3_, 300 MHz): δ 7.66 (1H, bs, NH), 7.44 (2H, d, J = 8.1 Hz, 2’-H, 6’-H), 7.40 (1H, dd, J = 5.1 Hz, 8.4 Hz, 7-H), 7.31 (2H, d, J = 8.4 Hz, 3’-H, 5’-H), 7.16 (1H, s, 10-H), 6.83 (1H, dd, J = 2.4 Hz, 8.7 Hz, 4-H), 6.60 (1H, td, J = 2.4 Hz, 9.0 Hz, 6-H), 3.57-3.47 (1H, m, NH-CH_2a_-CH_2_),3.49 (2H, s, 3-CH_2_), 3.22-3.13 (1H, m, NH-CH_2b_-CH_2_), 2.55 (3H, s, SCH_3_), 2.53-2.49 (1H, m, 5’’-H_a_), 2.17 (3H, s, 2-CH_3_), 2.16-2.13 (1H, m, 5’’-H_b_), 1.90 (3H, s, 1’’-NCH_3_), 1.86-1.33 (7H, m, 2’’-H, 3’’-H, 4’’-H, NH-CH_2_-CH_2_). HRMS calcd for [C_27_H_31_FN_2_OS+H]^+^: 451.22139, Found: 451.22101.

**(S,Z)-N-((1-Ethylpyrrolidin-2-yl)methyl)-2-(5-fluoro-2-methyl-1-(4-(methylthio)benzylidene)-1H-inden-3-yl)acetamide (28)**

By following method 1, the title compound **28** was obtained as a yellow solid in 97% (LCMS purity: 98.9%) yield. mp 74-76 ^o^C. ESI-MS m/z: 451.27 [M+H]^+^. ^1^H NMR (CDCl_3_, 300 MHz): δ 7.43 (2H, d, J = 8.1 Hz, 2’-H, 6’-H), 7.38 (1H, dd, J = 5.4 Hz, 8.7 Hz, 7-H), 7.30 (2H, d, J = 8.1 Hz, 3’-H, 5’-H), 7.16 (1H, s, 10-H), 6.86 (1H, dd, J = 2.4 Hz, 8.7 Hz, 4-H), 6.59 (1H, td, J = 2.7 Hz, 9.3 Hz, 6-H), 6.45 (1H, bs, NH), 3.52 (2H, s, 3-CH_2_), 3.42-3.34 (1H, m, NH-CH_2a_), 3.10-3.03 (1H, m, NH-CH_2b_), 2.88 (1H, bs, 2’’-H), 2.61--2.50 (2H, m, 1’’-NCH_2a_-CH_3_, 5’’-H_a_), 2.55 (3H, s, SCH_3_), 2.20 (3H, s, 2-CH_3_), 2.10--1.99 (2H, m, 1’’-NCH_2b_-CH_3_, 5’’-H_b_), 1.75--1.30 (4H, m, 3’’-H, 4’’-H), 0.86 (3H, t, J = 7.2 Hz, 1’’-NCH_2_-CH_3_). HRMS calcd for [C_27_H_31_FN_2_OS+H]^+^: 451.22139, Found: 451.22230.

**(S,Z)-2-(5-Fluoro-2-methyl-1-(4-(methylthio)benzylidene)-1H-inden-3-yl)-1-(2-(pyrrolidin-1-ylmethyl)pyrrolidin-1-yl)ethanone (29)**

By following method 1, the title compound **29** was obtained as a yellow solid in 93% (LCMS purity: 99.7%) yield. mp 101-104 ^o^C. ESI-MS m/z: 477.25 [M+H]^+^. ^1^H NMR (CDCl_3_, 300 MHz): δ 7.44 (2H, d, J = 8.1 Hz, 2’-H, 6’-H), 7.37 (1H, dd, J = 5.1 Hz, 8.1 Hz, 7-H), 7.28 (2H, d, J = 8.1 Hz, 3’-H, 5’-H), 7.14 (1H, s, 10-H), 6.84 (1H, dd, J = 2.4 Hz, 9.0 Hz, 4-H), 6.58 (1H, td, J = 2.1 Hz, 9.0 Hz, 6-H), 4.32-4.22 (1H, m, 2’’-H), 3.76-3.11 (10H, m, 3-CH_2_, 5’’-H, 2’’-CH_2_, 2’’’-H, 5’’’-H), 2.54 (3H, s, SCH_3_), 2.20 (3H, s, 2-CH_3_), 2.17-1.83 (8H, m, 3’’-H, 4’’-H, 3’’’-H, 4’’’-H). HRMS calcd for [C_29_H_33_FN_2_OS+H]^+^: 477.23704, Found: 477.23730.

**(Z)-1-([1,4'-Bipiperidin]-1'-yl)-2-(5-fluoro-2-methyl-1-(4-(methylthio)benzylidene)-1H-inden-3-yl)ethanone (30)**

By following method 1, the title compound **30** was obtained as a yellow solid in 97% (LCMS purity: 97.3%) yield. mp 145-148 ^o^C. ESI-MS m/z: 491.32 [M+H]^+^. ^1^H NMR (CDCl_3_, 300 MHz): δ 7.43 (2H, d, J = 8.1 Hz, 2’-H, 6’-H), 7.34 (1H, dd, J = 5.1 Hz, 8.4 Hz, 7-H), 7.28 (2H, d, J = 8.4 Hz, 3’-H, 5’-H), 7.10 (1H, s, 10-H), 6.93 (1H, dd, J = 2.4 Hz, 9.3 Hz, 4-H), 6.56 (1H, td, J = 2.4 Hz, 9.0 Hz, 6-H), 4.68 (1H, d, J = 12.9 Hz, 2’’-H_a_), 3.91 (1H, d, J = 13.5 Hz, 6’’-H_a_), 3.61 (1H, s, 3-CH_2a_), 3.59 (1H, s, 3-CH_2b_), 3.05-2.96 (1H, m, 6’’-H_b_), 2.59-2.38 (6H, m, 2’’-H_b_, 4’’-H, 2’’’-H, 6’’’-H), 2.54 (3H, s, SCH_3_), 2.17 (3H, s, 2-CH_3_), 1.85-1.29 (10H, m, 3’’-H, 5’’-H, 3’’’-H, 4’’’-H, 5’’’-H). HRMS calcd for [C_30_H_35_FN_2_OS+H]^+^: 491.25269, Found: 491.25281.

**(Z)-1-(4-(3-(Dimethylamino)propyl)piperazin-1-yl)-2-(5-fluoro-2-methyl-1-(4-(methylthio)benzylidene)-1H-inden-3-yl)ethanone** **(31)**

By following method 1, the title compound **31** was obtained as a yellow solid in 96% (LCMS purity: 93.9%) yield. mp 131-134 ^o^C. ESI-MS m/z: 494.28 [M+H]^+^. ^1^H NMR (DMSO, 300 MHz): δ 7.49 (2H, d, J = 8.1 Hz, 2’-H, 6’-H), 7.36 (2H, d, J = 8.4 Hz, 3’-H, 5’-H), 7.33 (1H, dd, *J* = 5.4 Hz, 8.7 Hz, 7-H), 7.26 (1H, s, 10-H), 6.98 (1H, dd, *J* = 2.4 Hz, 9.6 Hz, 4-H), 6.72 (1H, td, *J* = 2.4 Hz, 9.3 Hz, 6-H), 3.67 (2H, s, 3-CH_2_), 3.53-3.44 (4H, m, 2’’-H, 6’’-H), 2.60 (2H, t, *J* = 6.6 Hz, CH_2_-CH_2_-CH_2_N(CH_3_)_2_), 2.53 (3H, s, SCH_3_), 2.42 (6H, s, N(CH_3_)_2_), 2.36 (2H, t, *J* = 6.3 Hz, CH_2_-CH_2_-CH_2_N(CH_3_)_2_), 2.31 (4H, t, *J* = 7.2 Hz, 3’’-H, 5’’-H), 2.12 (3H, s, 2-CH_3_), 1.70-1.61 (2H, m, CH_2_-CH_2_-CH_2_N(CH_3_)_2_). HRMS calcd for [C_29_H_36_FN_3_OS+H]^+^: 494.26359, Found: 494.26345.

**(Z)-2-(5-Fluoro-2-methyl-1-(4-(methylthio)benzylidene)-1H-inden-3-yl)-1-(4-(pyridin-4-yl)piperazin-1-yl)ethanone (32)**

By following method 1, the title compound **32** was obtained as a yellow solid in 95% (LCMS purity: 100%) yield. mp 171-174 ^o^C. ESI-MS m/z: 486.14 [M+H]^+^. ^1^H NMR (DMSO, 300 MHz): δ 8.17 (2H, dd *J* = 1.5 Hz, 5.1 Hz, 2’’’-H, 6’’’-H), 7.50 (2H, d, J = 8.1 Hz, 2’-H, 6’-H), 7.36 (2H, d, J = 8.4 Hz, 3’-H, 5’-H), 7.33 (1H, dd, *J* = 5.1 Hz, 8.4 Hz, 7-H), 7.27 (1H, s, 10-H), 7.02 (1H, dd, *J* = 2.4 Hz, 9.3 Hz, 4-H), 6.84 (2H, dd *J* = 1.5 Hz, 5.1 Hz, 3’’’-H, 5’’’-H), 6.71 (1H, td, *J* = 2.7 Hz, 9.3 Hz, 6-H), 3.74 (2H, s, 3-CH_2_), 3.72-3.34 (8H, m, 2’’-H, 3’’-H, 5’’-H, 6’’-H), 2.53 (3H, s, SCH_3_), 2.13 (3H, s, 2-CH_3_). HRMS calcd for [C_29_H_28_FN_3_OS+H]^+^: 486.20099, Found: 486.20142.

**(Z)-N-(3-(1H-Imidazol-1-yl)propyl)-2-(5-fluoro-2-methyl-1-(4-(methylthio)benzylidene)-1H-inden-3-yl)acetamide (33)**

By following method 1, the title compound **33** was obtained as a yellow solid in 94% yield. mp 103-105 ^o^C. ESI-MS m/z: 448.26 [M+H]^+^. ^1^H NMR (DMSO, 300 MHz): δ 7.46 (2H, d, J = 8.4 Hz, 2’-H, 6’-H), 7.43 (1H, dd, J = 5.1 Hz, 8.4 Hz, 7-H), 7.32 (1H, s, 2’’-H), 7.30 (2H, d, J = 8.7 Hz, 3’-H, 5’-H), 7.20 (1H, s, 10-H), 7.01 (1H, s, 4’’-H), 6.84 (1H, dd, J = 2.1 Hz, 8.7 Hz, 4-H), 6.81 (1H, s, 5’’-H), 6.64 (1H, td, J = 2.4 Hz, 9.0 Hz, 6-H), 5.65 (1H, bt, CONH), 3.87 (2H, t, J = 6.9 Hz, CH_2_-Im), 3.53 (2H, s, 3-CH_2_), 3.20 (2H, q, J = 6.6 Hz, NH-CH_2_-CH_2_), 2.55 (3H, s, SCH_3_), 2.20 (3H, s, 2-CH_3_), 1.97-1.88 (2H, m, NH-CH_2_-CH_2_). HRMS calcd for [C_26_H_26_FN_3_OS+H]^+^: 448.18534, Found: 448.18591.

**(Z)-N-Benzyl-2-(5-fluoro-2-methyl-1-(4-(methylthio)benzylidene)-1H-inden-3-yl)acetamide (34)**

By following method 1, the title compound **34** was obtained as a yellow solid in 97% yield. mp 171-174 ^o^C. ESI-MS m/z: 430.17 [M+H]^+^.^1^H NMR (CDCl_3_, 300 MHz): δ 7.44 (2H, d, J = 8.1 Hz, 2’-H, 6’-H), 7.40 (1H, dd, J = 5.1 Hz, 8.4 Hz, 7-H), 7.29-7.13 (8H, m, 3’-H, 5’-H, Ph-H, 10-H), 6.86 (1H, dd, J = 2.4 Hz, 9.0 Hz, 4-H), 6.61 (1H, td, J = 2.4 Hz, 9.0 Hz, 6-H), 5.91 (1H, bs, NH), 4.42 (2H, d, J = 5.7 Hz, CH_2_-Ph), 3.59 (2H, s, 3-CH_2_), 2.54 (3H, s, SCH_3_), 2.19 (3H, s, 2-CH_3_). Anal. calcd for [C_27_H_24_FNOS]: C, 75.50; H, 5.63; N, 3.26. Found: C, 75.30; H, 5.81; N, 3.02.

**(Z)-N-(4-(Dimethylamino)benzyl)-2-(5-fluoro-2-methyl-1-(4-(methylthio)benzylidene)-1H-inden-3-yl)acetamide (35)**

By following method 1, the title compound **35** was obtained as a yellow solid in 98% yield. mp 174-177 ^o^C. ESI-MS m/z: 473.26 [M+H]^+^.^1^H NMR (CDCl_3_, 300 MHz): δ 7.45 (2H, d, J = 8.1 Hz, 3’-H, 5’-H), 7.38 (1H, dd, J = 5.1 Hz, 8.4 Hz, 7-H), 7.30 (2H, d, J = 8.4 Hz, 2’-H, 6’-H), 7.13 (1H, s, 10-H), 7.05 (2H, d, J = 8.7 Hz, 2’’-H, 6’’-H), 6.87 (1H, dd, J = 2.4 Hz, 9.0 Hz, 4-H), 6.64 (2H, d, J = 8.7 Hz, 3’’-H, 5’’-H), 6.59 (1H, td, J = 2.4 Hz, 8.4 Hz, 6-H), 5.76 (1H, bs, NH), 4.32 (2H, d, J = 5.7 Hz, CH_2_-Ar), 3.56 (2H, s, 3-CH_2_), 2.90 (6H, s, 4’’-N(CH_3_)_2_), 2.54 (3H, s, SCH_3_), 2.17 (3H, s, 2-CH_3_). Anal. calcd for [C_29_H_29_FN_2_OS]: C, 73.70; H, 6.18; N, 5.93. Found: C, 73.70; H, 6.17; N, 5.71.

**(Z)-2-(5-Fluoro-2-methyl-1-(4-(methylthio)benzylidene)-1H-inden-3-yl)-N-(furan-2-ylmethyl)acetamide (36)**

By following method 1, the title compound **36** was obtained as a yellow solid in 97% yield. mp 166-168 ^o^C. ESI-MS m/z: 420.19 [M+H]^+^.^1^H NMR (CDCl_3_, 300 MHz): δ 7.45 (2H, d, J = 8.1 Hz, 3’-H, 5’-H), 7.40 (1H, dd, J = 5.1 Hz, 8.4 Hz, 7-H), 7.30 (2H, d, J = 8.4 Hz, 2’-H, 6’-H), 7.29 (1H, dd, J = 0.9 Hz, 1.8 Hz, 5’’-H), 7.17 (1H, s, 10-H), 6.82 (1H, dd, J = 2.4 Hz, 8.7 Hz, 4-H), 6.61 (1H, td, J = 2.4 Hz, 9.3 Hz, 6-H), 6.27 (1H, dd, J = 1.8 Hz, 3.3 Hz, 4’’-H), 6.13 (1H, dd, J = 0.6 Hz, 3.3 Hz, 3’’-H), 5.91 (1H, bs, NH), 4.41 (2H, d, J = 5.7 Hz, CH_2_-Fu), 3.55 (2H, s, 3-CH_2_), 2.55 (3H, s, SCH_3_), 2.18 (3H, s, 2-CH_3_). Anal. calcd for [C_25_H_22_FNO_2_S]: C, 71.58; H, 5.29; N, 3.34. Found: C, 71.60; H, 5.09; N, 3.44.

**(S,Z)-Methyl 2-(2-(5-fluoro-2-methyl-1-(4-(methylthio)benzylidene)-1H-inden-3-yl)acetamido)propanoate (37)**

By following method 1, the title compound **37** was obtained as a yellow solid in 99% yield. mp 151-154 ^o^C. ESI-MS m/z: 426.22 [M+H]^+^. ^1^H NMR (CDCl_3_, 300 MHz): δ 7.46 (2H, d, J = 8.1 Hz, 3’-H, 5’-H), 7.41 (1H, dd, J = 5.4 Hz, 8.4 Hz, 7-H), 7.30 (2H, d, J = 8.4 Hz, 2’-H, 6’-H), 7.18 (1H, s, 10-H), 6.85 (1H, dd, J = 2.4 Hz, 8.7 Hz, 4-H), 6.61 (1H, td, J = 2.7 Hz, 9.3 Hz, 6-H), 6.13 (1H, d, J = 7.8 Hz, NH), 4.63-4.58 (1H, m, -CH), 3.70 (3H, s, OCH_3_), 3.54 (2H, s, 3-CH_2_), 2.55 (3H, s, SCH_3_), 2.22 (3H, s, 2-CH_3_), 1.34 (3H, d, J = 7.2 Hz, CH-CH_3_). Anal. calcd for [C_24_H_24_FNO_3_S]: C, 67.74; H, 5.68; N, 3.29. Found: C, 68.01; H, 5.70; N, 3.05.

**(Z)-2-(1-(Benzo[d]thiazol-2-ylmethylene)-5-fluoro-2-methyl-1H-inden-3-yl)-N-(2-(dimethylamino)ethyl)acetamide (38)**

By following method 2, the title compound **38** was obtained as a yellow solid in 53% (HPLC purity: 97.7%) yield. mp 132-135 ^o^C. ^1^H NMR (CDCl_3_, 400 MHz): δ 9.06 (1H, dd, J = 5.6 Hz, 8.4 Hz, 7-H), 8.18 (1H, d, J = 8.4 Hz, 4’-H), 7.94 (1H, d, J = 8.0 Hz, 7’-H), 7.56 (1H, td, J = 1.2 Hz, 7.2 Hz, 6’-H), 7.45 (1H, td, J = 1.2 Hz, 8.4 Hz, 5’-H), 7.07 (1H, s, 10-H), 6.89-6.81 (2H, m, 4-H, 6-H), 6.21 (1H, bs, NH), 3.51 (2H, s, 3-CH_2_), 3.28 (2H, q, J = 5.6 Hz, CONH-CH_2_), 2.32 (2H, t, J = 6.0 Hz, CH_2_N(CH_3_)_2_), 2.21 (3H, s, 2-CH_3_), 2.12 (6H, s, N(CH_3_)_2_). HRMS calcd for [C_24_H_24_FN_3_OS+H]^+^: 422.16969, Found: 422.17013.

**(Z)-2-(1-(Benzo[d]thiazol-2-ylmethylene)-5-fluoro-2-methyl-1H-inden-3-yl)-N-(2-(dimethylamino)ethyl)-N-methylacetamide (39)**

By following method 2, the title compound **39** was obtained as a yellow solid in 65% (HPLC purity: 98.7%) yield. mp 103-106 ^o^C. ^1^H NMR (CDCl_3_, 400 MHz): δ 8.98 (1H, dd, J = 5.2 Hz, 8.4 Hz, 7-H), 8.16 (1H, d, J = 8.4 Hz, 4’-H), 7.93 (1H, d, J = 8.0 Hz, 7’-H), 7.55 (1H, td, J = 1.2 Hz, 8.0 Hz, 6’-H), 7.44 (1H, t, J = 8.0 Hz, 5’-H), 7.02 (1H, s, 10-H), 6.94 (1H, dd, J = 2.8 Hz, 9.2 Hz, 4-H), 6.79 (1H, td, J = 2.4 Hz, 8.8 Hz, 6-H), 3.60 (2H, s, 3-CH_2_), 3.51 (2H, t, J = 6.8 Hz, CO(CH_3_)-CH_2_), 3.08 (3H, s, CO(CH_3_)-CH_2_), 2.46 (2H, t, J = 6.8 Hz, CH_2_N(CH_3_)_2_), 2.24 (6H, s, N(CH_3_)_2_), 2.18 (3H, s, 2-CH_3_). HRMS calcd for [C_25_H_26_FN_3_OS+H]^+^: 436.18534, Found: 436.18603.

**(Z)-2-(1-(Benzo[d]thiazol-2-ylmethylene)-5-fluoro-2-methyl-1H-inden-3-yl)-N-(2-(diethylamino)ethyl)acetamide (40)**

By following method 2, the title compound **40** was obtained as a yellow solid in 67% (HPLC purity: 96.2%) yield. mp 127-130 ^o^C. ^1^H NMR (CDCl_3_, 400 MHz): δ 9.08 (1H, dd, J = 5.2 Hz, 8.0 Hz, 7-H), 8.18 (1H, d, J = 8.0 Hz, 4’-H), 7.94 (1H, d, J = 8.0 Hz, 7’-H), 7.56 (1H, td, J = 1.2 Hz, 7.2 Hz, 6’-H), 7.45 (1H, td, J = 1.2 Hz, 8.0 Hz, 5’-H), 7.07 (1H, s, 10-H), 6.88-6.81 (2H, m, 4-H, 6-H), 6.38 (1H, bs, NH), 3.52 (2H, s, 3-CH_2_), 3.22 (2H, q, J = 5.6 Hz, CONH-CH_2_), 2.41 (2H, t, J = 6.0 Hz, CH_2_N(CH_2_-CH_3_)_2_), 2.32 (4H, q, J = 7.2 Hz, N(CH_2_-CH_3_)_2_), 2.21 (3H, s, 2-CH_3_), 0.77 (6H, t, J = 6.8 Hz, N(CH_2_-CH_3_)_2_). HRMS calcd for [C_26_H_28_FN_3_OS+H]^+^: 450.20099, Found: 450.20152.

**(Z)-2-(1-(Benzo[d]thiazol-2-ylmethylene)-5-fluoro-2-methyl-1H-inden-3-yl)-N-(2-(pyrrolidin-1-yl)ethyl)acetamide (41)**

By following method 2, the title compound **41** was obtained as a yellow solid in 54% (LCMS purity: 94.0%) yield. mp 162-165 ^o^C. ^1^H NMR (CDCl_3_, 400 MHz): δ 9.07 (1H, dd, J = 5.6 Hz, 8.4 Hz, 7-H), 8.18 (1H, d, J = 8.4 Hz, 4’-H), 7.94 (1H, d, J = 8.0 Hz, 7’-H), 7.57 (1H, td, J = 1.2 Hz, 7.2 Hz, 6’-H), 7.45 (1H, td, J = 0.8 Hz, 8.0 Hz, 5’-H), 7.07 (1H, s, 10-H), 6.88-6.82 (2H, m, 4-H, 6-H), 6.31 (1H, bs, NH), 3.52 (2H, s, 3-CH_2_), 3.29 (2H, q, J = 6.0 Hz, CONH-CH_2_), 2.49 (2H, t, J = 6.0 Hz, CH_2_-CH_2_N), 2.35 (4H, bs, 2’’-H, 5’’-H), 2.21 (3H, s, 2-CH_3_), 1.60 (4H, bs, 3’’-H, 4’’-H). HRMS calcd for [C_24_H_24_FN_3_OS+H]^+^: 422.16969, Found: 422.17013.

**(S,Z)-2-(1-(Benzo[d]thiazol-2-ylmethylene)-5-fluoro-2-methyl-1H-inden-3-yl)-N-((1-ethylpyrrolidin-2-yl)methyl)acetamide (42)**

By following method 2, the title compound **42** was obtained as a yellow solid in 53% (LCMS purity: 95.3%) yield. mp 168-171 ^o^C. ^1^H NMR (CDCl_3_, 400 MHz): δ 9.10 (1H, dd, J = 5.2 Hz, 8.4 Hz, 7-H), 8.18 (1H, d, J = 8.4 Hz, 4’-H), 7.94 (1H, d, J = 8.0 Hz, 7’-H), 7.56 (1H, td, J = 1.2 Hz, 7.2 Hz, 6’-H), 7.45 (1H, td, J = 1.2 Hz, 8.4 Hz, 5’-H), 7.07 (1H, s, 10-H), 6.90-6.82 (2H, m, 4-H, 6-H), 6.50 (1H, bs, NH), 3.60 (2H, s, 3-CH_2_), 3.45-3.38 (1H, m, NH-CH_2a_), 3.12-3.04 (1H, m, NH-CH_2b_), 2.96-2.88 (1H, bs, 2’’-H), 2.61-2.54 (2H, m, 1’’-NCH_2a_-CH_3_, 5’’-H_a_), 2.23 (3H, s, 2-CH_3_), 2.13-2.00 (2H, m, 1’’-NCH_2b_-CH_3_, 5’’-H_b_), 1.80-1.35 (4H, m, 3’’-H, 4’’-H), 0.87 (3H, t, J = 6.4 Hz, 1’’-NCH_2_-CH_3_). HRMS calcd for [C_27_H_28_FN_3_OS+H]^+^: 462.20099, Found: 462.20176.

**(S,Z)-2-(1-(Benzo[d]thiazol-2-ylmethylene)-5-fluoro-2-methyl-1H-inden-3-yl)-1-(2-(pyrrolidin-1-ylmethyl)pyrrolidin-1-yl)ethanone (43)**

By following method 2, the title compound **43** was obtained as a yellow solid in 52% (HPLC purity: 92.9%) yield. mp 79-81 ^o^C. ^1^H NMR (CDCl_3_, 400 MHz): δ 8.99 (1H, dd, J = 5.6 Hz, 8.8 Hz, 7-H), 8.16 (1H, d, J = 8.0 Hz, 4’-H), 7.93 (1H, d, J = 8.0 Hz, 7’-H), 7.55 (1H, td, J = 1.2 Hz, 7.2 Hz, 6’-H), 7.44 (1H, td, J = 1.2 Hz, 8.4 Hz, 5’-H), 7.03 (1H, s, 10-H), 6.91 (1H, dd, J = 2.0 Hz, 8.8 Hz, 4-H), 6.80 (1H, td, J = 2.8 Hz, 8.8 Hz, 6-H), 4.33-4.26 (1H, m, 2’’-H), 3.79-2.58 (10H, m, 5’’-H, 2’’-CH_2_, 2’’’-H, 5’’’-H, 3-CH_2_), 2.20 (3H, s, 2-CH_3_), 2.11-1.78 (8H, m, 3’’-H, 4’’-H, 3’’’-H, 4’’’-H). HRMS calcd for [C_29_H_30_FN_3_OS+H]^+^: 488.21664, Found: 488.21676.

**(Z)-N-(2-(Dimethylamino)ethyl)-2-(5-fluoro-2-methyl-1-(4-(methylthio)benzylidene)-1H-inden-3-yl)propanamide (44)**

By following methods 2-5, the title compound **44** was obtained as a yellow viscous liquid in 91% (HPLC purity: 100%) yield in the final step. ^1^H NMR (CDCl_3_, 400 MHz): δ 7.43 (2H, d, J = 8.0 Hz, 2’-H, 6’-H), 7.37 (1H, dd, J = 5.2 Hz, 8.4 Hz, 7-H), 7.29 (2H, d, J = 8.4 Hz, 3’-H, 5’-H), 7.15 (1H, s, 10-H), 6.91 (1H, dd, J = 2.4 Hz, 9.2 Hz, 4-H), 6.57 (1H, td, J = 2.8 Hz, 9.2 Hz, 6-H), 6.14 (1H, s, NH), 3.81 (1H, q, J = 7.2 Hz, 3-C(CH_3_)H), 3.34 (2H, q, J = 5.6 Hz, CO-NHCH_2_), 2.55 (3H, s, SCH_3_), 2.47 (2H, t, J = 5.6 Hz, CO-NHCH_2_CH_2_), 2.23 (6H, s, -N(CH_3_)_2_), 2.20 (3H, s, 2-CH_3_), 1.42 (3H, d, J = 7.2 Hz, 3-C(CH_3_)H). HRMS calcd for [C_25_H_29_FN_2_OS+H]^+^: 425.20574, Found: 425.20689.

**(Z)-N-(2-(Dimethylamino)ethyl)-2-(5-fluoro-2-methyl-1-(4-(methylthio)benzylidene)-1H-inden-3-yl)-N-methylpropanamide (45)**

By following methods 2-5, the title compound **45** was obtained as a yellow viscous liquid in 92% (HPLC purity: 100%) yield in the final step. ^1^H NMR (CDCl_3_, 400 MHz): δ 7.44 (2H, d, J = 8.4 Hz, 2’-H, 6’-H), 7.37 (1H, dd, J = 5.2 Hz, 8.4 Hz, 7-H), 7.29 (2H, d, J = 8.4 Hz, 3’-H, 5’-H), 7.09 (1H, s, 10-H), 7.05 (1H, dd, J = 2.4 Hz, 8.8 Hz, 4-H), 6.58 (1H, td, J = 2.4 Hz, 8.8 Hz, 6-H), 3.86 (1H, q, J = 6.8 Hz, 3-C(CH_3_)H), 3.52-3.15 (2H, m, CO-N(CH_3_)CH_2_), 2.86 (3H, s, CO-N(CH_3_)CH_2_), 2.55 (3H, s, SCH_3_), 2.39 (2H, t, J = 6.8 Hz, CO-N(CH_3_)-CH_2_-CH_2_), 2.23 (3H, s, -N(CH_3_)_2_), 2.18 (3H, s, 2-CH_3_), 2.08 (3H, s, -N(CH_3_)_2_), 1.44 (3H, d, J = 7.2 Hz, 3-C(CH_3_)H). HRMS calcd for [C_26_H_31_FN_2_OS+H]^+^: 439.22139, Found: 439.22225.

**(Z)-N-(2-(Diethylamino)ethyl)-2-(5-fluoro-2-methyl-1-(4-(methylthio)benzylidene)-1H-inden-3-yl)propanamide (46)**

By following methods 2-5, the title compound **46** was obtained as a yellow solid in 95% (HPLC purity: 92.3%) yield in the final step. mp 75-77 ^o^C. ^1^H NMR (CDCl_3_, 400 MHz): δ 7.42 (2H, d, J = 8.4 Hz, 2’-H, 6’-H), 7.36 (1H, dd, J = 5.6 Hz, 8.4 Hz, 7-H), 7.28 (2H, d, J = 8.4 Hz, 3’-H, 5’-H), 7.19 (1H, s, 10-H), 6.91 (1H, dd, J = 2.4 Hz, 9.6 Hz, 4-H), 6.88 (1H, bs, NH), 6.56 (1H, td, J = 2.8 Hz, 9.2 Hz, 6-H), 3.93 (1H, q, J = 6.8 Hz, 3-C(CH_3_)H), 3.55-3.45 (2H, m, CONH-CH_2_), 3.25-3.00 (2H, m, CH_2_-CH_2_N(CH_2_-CH_3_)_2_), 3.08 (4H, q, J = 6.8 Hz, -N(CH_2_-CH_3_)_2_), 2.54 (3H, s, SCH_3_), 2.23 (3H, s, 2-CH_3_), 1.48 (3H, d, J = 7.2 Hz, 3-C(CH_3_)H), 1.26 (6H, t, J = 7.2 Hz, -N(CH_2_-CH_3_)_2_). HRMS calcd for [C_27_H_33_FN_2_OS+H]^+^: 453.23704, Found: 453.23827.

**(Z)-N-(3-(Dibutylamino)propyl)-2-(5-fluoro-2-methyl-1-(4-(methylthio)benzylidene)-1H-inden-3-yl)propanamide (47)**

By following methods 2-5, the title compound **47** was obtained as a yellow solid in 86% yield in the final step. mp 121-124 ^o^C. ^1^H NMR (CDCl_3_, 400 MHz): δ 7.42 (2H, d, J = 8.0 Hz, 2’-H, 6’-H), 7.36 (1H, dd, J = 5.6 Hz, 8.8 Hz, 7-H), 7.28 (2H, d, J = 8.4 Hz, 3’-H, 5’-H), 7.18 (1H, s, 10-H), 6.96 (1H, dd, J = 2.4 Hz, 9.6 Hz, 4-H), 6.77 (1H, bs, NH), 6.56 (1H, td, J = 2.4 Hz, 9.2 Hz, 6-H), 3.92 (1H, q, J = 7.2 Hz, 3-C(CH_3_)H), 3.32 (2H, q, J = 5.6 Hz, CONH-CH_2_), 2.90-2.70 (6H, m, CH_2_N(CH_2_(CH_2_)_2_CH_3_)_2_), 2.54 (3H, s, SCH_3_), 2.24 (3H, s, 2-CH_3_), 1.94-1.78 (2H, m, CH_2_-CH_2_N(Bu)_2_), 1.57-1.25 (8H, m, N(CH_2_(CH_2_)_2_CH_3_)_2_), 1.48 (3H, d, J = 7.2 Hz, 3-C(CH_3_)H), 0.93 (6H, t, J = 7.2 Hz, N(CH_2_(CH_2_)_2_CH_3_)_2_). HRMS calcd for [C_32_H_43_FN_2_OS+H]^+^: 523.31529, Found: 523.31552.

**(Z)-2-(5-Fluoro-2-methyl-1-(4-(methylthio)benzylidene)-1H-inden-3-yl)-N-methyl-N-(2-(methylamino)ethyl)propanamide (48)**

By following methods 2-5, the title compound **48** was obtained as a yellow viscous liquid in 68% (HPLC purity: 99.1%) yield in the final step. ^1^H NMR (CDCl_3_, 400 MHz): δ 7.43 (2H, d, J = 8.0 Hz, 2’-H, 6’-H), 7.36 (1H, dd, J = 5.2 Hz, 8.4 Hz, 7-H), 7.28 (2H, d, J = 8.4 Hz, 3’-H, 5’-H), 7.11 (1H, s, 10-H), 7.10 (1H, dd, J = 2.4 Hz, 9.6 Hz, 4-H), 6.57 (1H, td, J = 2.4 Hz, 8.8 Hz, 6-H), 3.88 (1H, q, J =6.8 Hz, 3-C(CH_3_)H), 3.64-3.35 (2H, m, CO-N(CH_3_)-CH_2_-CH_2_), 2.86 (3H, s, CO-N(CH_3_)-), 2.77-2.65 (2H, m, CO-N(CH_3_)-CH_2_-CH_2_), 2.54 (3H, s, SCH_3_), 2.39 (3H, s, -N(CH_3_)H), 2.18 (3H, s, 2-CH_3_), 1.43 (3H, d, J = 6.8 Hz, 3-C(CH_3_)H). HRMS calcd for [C_25_H_29_FN_2_OS+H]^+^: 425.20574, Found: 425.20627.

**(Z)-2-(5-Fluoro-2-methyl-1-(4-(methylthio)benzylidene)-1H-inden-3-yl)-N-(2-(piperidin-1-yl)ethyl)propanamide (49)**

By following methods 2-5, the title compound **49** was obtained as a yellow viscous liquid in 90% (HPLC purity: 99.1%) yield in the final step. ^1^H NMR (CDCl_3_, 400 MHz): δ 7.42 (2H, d, J = 8.4 Hz, 2’-H, 6’-H), 7.35 (1H, dd, J = 5.6 Hz, 8.4 Hz, 7-H), 7.29 (2H, d, J = 8.4 Hz, 3’-H, 5’-H), 7.15 (1H, s, 10-H), 6.93 (1H, dd, J = 2.0 Hz, 9.2 Hz, 4-H), 6.56 (1H, td, J = 2.4 Hz, 8.8 Hz, 6-H), 6.49 (1H, bs, NH), 3.79 (1H, q, J = 7.2 Hz, 3-C(CH_3_)H), 3.38-3.23 (2H, m, NH-CH_2_-), 2.55 (3H, s, SCH_3_), 2.44-2.17 (6H, m, 2’’-H, 6’’-H, NH-CH_2_-CH_2_), 2.22 (3H, s, 2-CH_3_), 1.51 (3H, d, J = 7.2 Hz, 3-C(CH_3_)H), 1.32 (6H, bs, 3’’-H, 4’’-H, 5’’-H). HRMS calcd for [C_28_H_33_FN_2_OS+H]^+^: 465.23704, Found: 465.23782.

**(Z)-2-(5-Fluoro-2-methyl-1-(4-(methylthio)benzylidene)-1H-inden-3-yl)-N-(3-(2-methylpiperidin-1-yl)propyl)propanamide (50)**

By following methods 2-5, the title compound **50** was obtained as a yellow solid in 88% (HPLC purity: 97.1%) yield in the final step. mp 87-89 ^o^C. ^1^H NMR (CDCl_3_, 400 MHz): δ 7.42 (2H, d, J = 8.4 Hz, 2’-H, 6’-H), 7.36 (1H, dd, J = 5.6 Hz, 8.8 Hz, 7-H), 7.29 (2H, d, J = 8.4 Hz, 3’-H, 5’-H), 7.19 (1H, s, 10-H), 6.93 (1H, dd, J = 2.0 Hz, 9.2 Hz, 4-H), 6.62 (1H, bs, NH), 6.567 (1H, td, J = 2.4 Hz, 8.8 Hz, 6-H), 3.97 (1H, q, J = 7.2 Hz, 3-C(CH_3_)H), 3.37-2.71 (7H, m, NH-CH_2_-CH_2_-CH_2_, 2’’-H, 6’’-H), 2.54 (3H, s, SCH_3_), 2.25 (3H, s, 2-CH_3_), 2.03-1.54 (8H, m, NH-CH_2_-CH_2_, 3’’-H, 4’’-H, 5’’-H), 1.50 (3H, d, J = 6.8 Hz, 3-C(CH_3_)H), 1.25 (3H, d, J = 6.4 Hz, 2’’-CH_3_). HRMS calcd for [C_30_H_37_FN_2_OS+H]^+^: 493.26834, Found: 493.26969.

**N-(((S)-1-Ethylpyrrolidin-2-yl)methyl)-2-((Z)-5-fluoro-2-methyl-1-(4-(methylthio)benzylidene)-1H-inden-3-yl)propanamide (51)**

By following methods 2-5, the title compound **51** was obtained as a yellow viscous liquid in 95% (HPLC purity: 98.9%) yield in the final step. ^1^H NMR (CDCl_3_, 400 MHz): δ 7.43 (2H, d, J = 8.4 Hz, 2’-H, 6’-H), 7.37-7.33 (1H, m, 7-H), 7.29 (2H, d, J = 8.8 Hz, 3’-H, 5’-H), 7.14 (1H, s, 10-H), 6.96-6.92 (1H, m, 4-H), 6.59-6.54 (1H, m, 6-H), 6.26 (1H, bs, NH), 3.83-3.76 (1H, m, 3-C(CH_3_)H), 3.48-3.29 (1H, m, NH-CH_2a_), 3.19-3.00 (1H, m, NH-CH_2b_), 2.88-2.81 (1H, bs, 2’’-H), 2.72-2.37 (1H, m, 1’’-NCH_2a_-CH_3_), 2.55 (3H, s, SCH_3_), 2.21 (3H, s, 2-CH_3_), 2.16-1.94 (3H, m, 1’’-NCH_2b_-CH_3_, 5’’-H), 1.83-1.41 (4H, m, 3’’-H, 4’’-H), 1.54 (3H, d, J = 7.2 Hz, 3-C(CH_3_)H), 0.97 (3H, t, J = 7.2 Hz, 1’’-NCH_2_-CH_3_). HRMS calcd for [C_28_H_33_FN_2_OS+H]^+^: 465.23704, Found: 465.23712.

**2-((Z)-5-Fluoro-2-methyl-1-(4-(methylthio)benzylidene)-1H-inden-3-yl)-1-((S)-2-(pyrrolidin-1-ylmethyl)pyrrolidin-1-yl)propan-1-one (52)**

By following methods 2-5, the title compound **52** was obtained as a yellow solid in 96% (HPLC purity: 99.9%) yield in the final step. mp 80-82 ^o^C. ^1^H NMR (CDCl_3_, 400 MHz): δ 7.45-7.26 (5H, m, 7-H, 2’-H, 3’-H, 5’-H, 6’-H), 7.14-7.08 (2H, m, 10-H, 4-H), 6.62-6.54 (1H, m, 6-H), 4.34-4.24 (1H, m, 2’’-H), 3.90-3.76 (1H, m, 3-C(CH_3_)H), 3.50-2.67 (8H, m, 5’’-H, 2’’-CH_2_, 2’’’-H, 5’’’-H), 2.55 (3H, s, SCH_3_), 2.17 (3H, s, 2-CH_3_), 1.94-1.71 (8H, m, 3’’-H, 4’’-H, 3’’’-H, 4’’’-H), 1.44 (3H, d, J = 6.8 Hz, 3-C(CH_3_)H). HRMS calcd for [C_30_H_35_FN_2_OS+H]^+^: 491.25269, Found: 491.25277.

**(Z)-1-([1,4'-Bipiperidin]-1'-yl)-2-(5-fluoro-2-methyl-1-(4-(methylthio)benzylidene)-1H-inden-3-yl)propan-1-one (53)**

By following methods 2-5, the title compound **53** was obtained as a yellow solid in 86% (HPLC purity: 100%) yield in the final step. mp 80-82 ^o^C. ^1^H NMR (CDCl_3_, 400 MHz): δ 7.42 (2H, d, J = 8.0 Hz, 2’-H, 6’-H), 7.34 (1H, dd, J = 5.2 Hz, 8.4 Hz, 7-H), 7.28 (2H, d, J = 8.4 Hz, 3’-H, 5’-H), 7.11 (1H, s, 10-H), 7.07 (1H, dd, J = 2.4 Hz, 8.8 Hz, 4-H), 6.56 (1H, td, J = 2.4 Hz, 8.8 Hz, 6-H), 4.80-4.68 (1H, m, 2’’-H_a_), 3.85 (1H, q, J = 7.2 Hz, 3-C(CH_3_)H), 3.67 (1H, d, J = 13.2 Hz, 6’’-H_a_), 2.89-2.66 (1H, m, 6’’-H_b_), 2.57-2.14 (6H, m, 2’’-H_b_, 4’’-H, 2’’’-H, 6’’’-H), 2.54 (3H, s, SCH_3_), 2.18 (3H, s, 2-CH_3_), 1.87-0.74 (10H, m, 3’’-H, 5’’-H, 3’’’-H, 4’’’-H, 5’’’-H), 1.43 (3H, d, J = 6.8 Hz, 3-C(CH_3_)H). HRMS calcd for [C_31_H_37_FN_2_OS+H]^+^: 505.26834, Found: 505.26937.

**(Z)-1-(4-(3-(Dimethylamino)propyl)piperazin-1-yl)-2-(5-fluoro-2-methyl-1-(4-(methylthio)benzylidene)-1H-inden-3-yl)propan-1-one (54)**

By following methods 2-5, the title compound **54** was obtained as a yellow solid in 91% (HPLC purity: 100%) yield in the final step. mp 78-80 ^o^C. ^1^H NMR (CDCl_3_, 400 MHz): δ 7.43 (2H, d, J = 8.0 Hz, 2’-H, 6’-H), 7.36 (1H, dd, J = 5.2 Hz, 8.4 Hz, 7-H), 7.28 (2H, d, J = 8.4 Hz, 3’-H, 5’-H), 7.11 (1H, s, 10-H), 7.01 (1H, dd, J = 2.4 Hz, 9.6 Hz, 4-H), 6.57 (1H, td, J = 2.4 Hz, 9.2 Hz, 6-H), 3.83 (1H, q, J = 6.8 Hz, 3-C(CH_3_)H), 3.79 (1H, bs, 2’’-H_a_), 3.54-3.47 (1H, m, 2’’-H_b_), 3.28 (2H, t, J = 4.8 Hz, 6’’-H), 2.56 (2H, t, J = 6.8 Hz, 4’’-N-CH_2_-CH_2_-CH_2_-N(CH_3_)_2_), 2.55 (3H, s, SCH_3_), 2.52-2.44 (1H, m, 3’’-H_a_), 2.42 (6H, s, N(CH_3_)_2_), 2.36-2.31 (1H, m, 5’’-H_a_), 2.32 (2H, t, J = 6.8 Hz, 4’’-N-CH_2_-CH_2_-CH_2_-N(CH_3_)_2_), 2.22-2.17 (1H, m, 3’’-H_b_), 2.15 (3H, s, 2-CH_3_), 1.97-1.90 (1H, m, 5’’-H_b_), 1.69-1.62 (2H, m, 4’’-N-CH_2_-CH_2_-CH_2_-N(CH_3_)_2_), 1.41 (3H, d, J = 7.2 Hz, 3-C(CH_3_)H). HRMS calcd for [C_30_H_38_FN_3_OS+H]^+^: 508.27924, Found: 508.28097.

**(Z)-2-(5-Fluoro-2-methyl-1-(4-(methylthio)benzylidene)-1H-inden-3-yl)-1-(4-(pyridin-4-yl)piperazin-1-yl)propan-1-one (55)**

By following methods 2-5, the title compound **55** was obtained as a yellow viscous liquid in 89% (HPLC purity: 100%) yield in the final step. ^1^H NMR (CDCl_3_, 400 MHz): δ 8.25 (2H, d, J = 4.8 Hz, 2’’’-H, 6’’’-H), 7.42 (2H, d, J = 8.0 Hz, 2’-H, 6’-H), 7.38 (1H, dd, J = 5.2 Hz, 8.4 Hz, 7-H), 7.28 (2H, d, J = 8.4 Hz, 3’-H, 5’-H), 7.13 (1H, s, 10-H), 7.04 (1H, dd, J = 2.4 Hz, 9.6 Hz, 4-H), 6.59 (1H, td, J = 2.4 Hz, 8.8 Hz, 6-H), 6.55 (2H, d, J = 5.2 Hz, 3’’’-H, 5’’’-H), 4.05-4.00 (1H, m, 2’’-H_a_), 3.86 (1H, q, J = 6.8 Hz, 3-C(CH_3_)H), 3.63-3.57 (1H, m, 2’’-H_b_), 3.48-3.36 (3H, m, 6’’-H, 3’’-H_a_), 3.28-2.79 (3H, m, 3’’-H_b_, 5’’-H), 2.54 (3H, s, SCH_3_), 2.19 (3H, s, 2-CH_3_), 1.46 (3H, d, J = 7.2 Hz, 3-C(CH_3_)H). HRMS calcd for [C_30_H_30_FN_3_OS+H]^+^: 500.21664, Found: 500.21765.

**(Z)-2-Amino-N-(2-(2-(5-fluoro-2-methyl-1-(4-(methylthio)benzylidene)-1H-inden-3-yl)-N-methylacetamido)ethyl)-N,3-dimethylbutanamide (56)**

By following method 1, the title compound **56** was obtained as a yellow viscous liquid in 93% (LCMS purity: 97.0%) yield. ESI-MS m/z: 510.31 [M+H]^+^.^1^H NMR (CDCl_3_, 300 MHz): δ 7.43 (2H, d, J = 8.4 Hz, 3’-H, 5’-H), 7.34 (1H, dd, J = 5.4 Hz, 8.7 Hz, 7-H), 7.28 (2H, d, J = 8.7 Hz, 2’-H, 6’-H), 7.10 (1H, s, 10-H), 6.91 (1H, dd, J = 2.4 Hz, 9.3 Hz, 4-H), 6.56 (1H, td, J = 2.4 Hz, 9.3 Hz, 6-H), 3.68-3.41 (7H, m, 3-CH_2_, CH_2_-CH_2_, CO-CH), 3.13 (3H, s, CO-N(CH_3_)-), 3.04 (3H, s, -N(CH_3_)-CO), 2.54 (3H, s, SCH_3_), 2.16 (3H, s, 2-CH_3_), 1.86-1.79 (1H, m, CH(CH_3_)_2_), 0.97 (3H, d, J = 6.9 Hz, CH(CH_3_)_2_), 0.90 (3H, d, J = 6.9 Hz, CH(CH_3_)_2_). HRMS calcd for [C_29_H_36_FN_3_O_2_S+H]^+^: 510.25850, Found: 510.25797.

**(2S)-2-Amino-N-(2-(2-((Z)-5-fluoro-2-methyl-1-(4-(methylthio)benzylidene)-1H-inden-3-yl)-N-methylpropanamido)ethyl)-N,3-dimethylbutanamide (57)**

By following methods 2-5, the title compound **57** was obtained as a yellow solid in 95% (HPLC purity: 92.3%) yield. mp 60-62 ^o^C. ^1^H NMR (DMSO, 400 MHz): δ 7.43 (2H, d, J = 8.0 Hz, 2’-H, 6’-H), 7.35-7.19 (5H, m, 7-H, 3’-H, 5’-H, 10-H, 4-H), 6.73 (1H, td, J = 2.0 Hz, 8.8 Hz, 6-H), 4.15-3.98 (1H, m, COCH), 3.67-2.78 (11H, m, CH_2_-CH_2_, 3-C(CH_3_)H, CO-N(CH_3_), N(CH_3_)-CO), 2.53 (3H, s, SCH_3_), 2.17 (3H, s, 2-CH_3_), 1.68-1.59 (1H, m, CH(CH_3_)_2_), 1.28-1.23 (3H, m, 3-C(CH_3_)H), 0.86-0.75 (6H, m, CH(CH_3_)_2_). HRMS calcd for [C_30_H_38_FN_3_O_2_S+H]^+^: 524.27415, Found: 524.27412.

**(Z)-N-Benzyl-2-(5-fluoro-2-methyl-1-(4-(methylthio)benzylidene)-1H-inden-3-yl)ethanamine (58)**

By following methods 3, 6 and 7, the title compound **58** was obtained as a yellow viscous liquid in 74% (LCMS purity: 100%) yield in the final step. ESI-MS m/z: 416.20 [M+H]^+^.^1^H NMR (CDCl_3_, 300 MHz): δ 7.43 (2H, d, J = 8.1 Hz, 3’-H, 5’-H), 7.35-7.21 (8H, m, 2’-H, 6’-H, 7-H, Ph-H), 7.06 (1H, s, 10-H), 6.82 (1H, dd, J = 2.4 Hz, 9.0 Hz, 4-H), 6.56 (1H, td, J = 2.4 Hz, 9.3 Hz, 6-H), 3.83 (2H, s, -CH_2_-Ph), 2.89-2.75 (4H, m, -CH_2_-CH_2_-NH), 2.54 (3H, s, -SCH_3_), 2.16 (3H, s, 2-CH_3_). HRMS calcd for [C_27_H_26_FNS+H]^+^: 416.18428, Found: 416.18452.

**(Z)-2-(5-Fluoro-2-methyl-1-(4-(methylthio)benzylidene)-1H-inden-3-yl)-N-(furan-2-ylmethyl)ethanamine (59)**

By following methods 3, 6 and 7, the title compound **59** was obtained as a yellow viscous liquid in 68% (LCMS purity: 95.8%) yield in the final step. ESI-MS m/z: 406.23 [M+H]^+^.^1^H NMR (CDCl_3_, 300 MHz): δ 7.44 (2H, d, J = 8.4 Hz, 3’-H, 5’-H), 7.36-7.26 (4H, m, 2’-H, 6’-H, 7-H, 5’’-H), 7.06 (1H, s, 10-H), 6.84 (1H, dd, J = 2.4 Hz, 9.0 Hz, 4-H), 6.59 (1H, td, J = 2.4 Hz, 9.3 Hz, 6-H), 6.31 (1H, dd, J = 1.8 Hz, 3.3 Hz, 4’’-H), 6.16 (1H, dd, J = 0.6 Hz, 3.0 Hz, 3’’-H), 3.82 (2H, s, -CH_2_-Furan), 2.86-2.73 (4H, m, -CH_2_-CH_2_-NH), 2.54 (3H, s, -SCH_3_), 2.16 (3H, s, 2-CH_3_). HRMS calcd for [C_25_H_24_FNOS+H]^+^: 406.16354, Found: 406.16388.

**(Z)-2-(5-Fluoro-2-methyl-1-(4-(methylthio)benzylidene)-1H-inden-3-yl)-N-(2-(piperidin-1-yl)ethyl)ethanamine (60)**

By following methods 3, 6 and 7, the title compound **60** was obtained as a yellow viscous liquid in 55% (LCMS purity: 94.6%) yield in the final step. ESI-MS m/z: 437.32 [M+H]^+^.^1^H NMR (CDCl_3_, 300 MHz): δ 7.43 (2H, d, J = 8.4 Hz, 3’-H, 5’-H), 7.36 (1H, dd, J = 5.4 Hz, 8.4 Hz, 7-H), 7.29 (2H, d, J = 8.4 Hz, 2’-H, 6’-H), 7.09 (1H, s, 10-H), 6.87 (1H, dd, J = 2.4 Hz, 9.0 Hz, 4-H), 6.60 (1H, td, J = 2.4 Hz, 9.0 Hz, 6-H), 2.88-2.77 (6H, m, -CH_2_-CH_2_-NH-CH_2_-), 2.54 (3H, s, -SCH_3_), 2.51 (2H, t, J = 6.0 Hz, NH-CH_2_-CH_2_), 2.38 (4H, t, J = 5.1 Hz, 2’’-H, 6’’-H), 2.19 (3H, s, 2-CH_3_), 1.51-1.48 (4H, m, 3’’-H, 5’’-H), 1.41-1.35 (2H, m, 4’’-H). HRMS calcd for [C_27_H_33_FN_2_S+H]^+^: 437.24212, Found: 437.24195.

**(E)-N-(2-(Dimethylamino)ethyl)-2-(5-fluoro-2-methyl-1-(4-(methylthio)benzylidene)-1H-inden-3-yl)acetamide (61)**

By following method 1, the title compound **61** was obtained as a yellow crystalline solid. mp 153-155 ^o^C. ESI-MS m/z: 411.20 [M+H]^+^.^1^H NMR (DMSO, 300 MHz): δ 8.02 (1H, t, J = 5.4 Hz, NH), 7.72 (1H, s, 10-H), 7.70 (1H, dd, J = 5.1 Hz, 8.4 Hz, 7-H), 7.38-7.28 (4H, m, 2’-H, 3’-H, 5’-H, 6’-H), 7.14 (1H, dd, J = 2.4 Hz, 9.6 Hz, 4-H), 6.92 (1H, td, J = 2.4 Hz, 9.9 Hz, 6-H), 3.41 (2H, s, 3-CH_2_), 3.14 (2H, q, J = 6.3 Hz, NH-CH_2_-CH_2_), 2.51 (3H, s, SCH_3_), 2.26 (2H, t, J = 6.6 Hz, NH-CH_2_-CH_2_), 2.16 (6H, s, N(CH_3_)_2_), 1.86 (3H, s, 2-CH_3_). Anal. calcd for [C_24_H_27_FN_2_OS]: C, 70.21; H, 6.63; N, 6.82. Found: C, 70.42; H, 6.70; N, 6.49.

**(E)-N-(2-(dimethylamino)ethyl)-2-(5-fluoro-1-(3,4,5-trimethoxybenzylidene)-1H-inden-3-yl)acetamide (62)**

By following method 1, the title compound **62** was obtained as a yellow solid. ESI-MS m/z: 441.28 [M+H]^+^. ^1^H NMR (CDCl_3_, 300 MHz): δ 7.60 (1H, dd, J = 4.8 Hz, 8.4 Hz, 7-H), 7.32 (1H, s, 10-H), 7.04 (1H, dd, J = 2.4 Hz, 9.0 Hz, 4-H), 7.01 (1H, s, 2-H), 6.94 (1H, td, J = 2.1 Hz, 8.4 Hz, 6-H), 6.81 (2H, s, 2’-H, 6’-H), 6.37 (1H, bs, NH), 3.92 (6H, s, 3’-OCH_3_, 5’-OCH_3_), 3.90 (3H, s, 4’-OCH_3_), 3.57 (2H, s, 3-CH_2_), 3.31 (2H, q, J = 5.7 Hz, CON(H)CH_2_), 2.36 (2H, t, J = 6.0 Hz, CH_2_N(CH_3_)), 2.13 (6H, s, N(CH_3_)_2_). Anal. calcd for [C_25_H_29_FN_2_O_4_]: C, 68.16; H, 6.64; N, 6.36. Found: C, 68.19; H, 6.39; N, 6.71.

**(E)-2-(5-fluoro-1-(3,4,5-trimethoxybenzylidene)-1H-inden-3-yl)-N-(2-(pyrrolidin-1-yl)ethyl)acetamide (63)**

By following method 1, the title compound **63** was obtained as a yellow solid. ESI-MS m/z: 467.24 [M+H]^+^. ^1^H NMR (CDCl_3_, 300 MHz): δ 7.60 (1H, dd, J = 4.8 Hz, 8.4 Hz, 7-H), 7.33 (1H, s, 10-H), 7.04 (1H, dd, J = 2.4 Hz, 9.0 Hz, 4-H), 7.01 (1H, s, 2-H), 6.94 (1H, td, J = 2.4 Hz, 9.0 Hz, 6-H), 6.81 (2H, s, 2’-H, 6’-H), 6.39 (1H, bs, NH), 3.92 (6H, s, 3’-OCH_3_, 5’-OCH_3_), 3.91 (3H, s, 4’-OCH_3_), 3.59 (2H, s, 3-CH_2_), 3.32 (2H, q, J = 5.7 Hz, NH-CH_2_-CH_2_), 2.53 (2H, t, J = 6.0 Hz, NH-CH_2_-CH_2_), 2.3 (4H, bs, 2’’-H, 5’’-H), 1.62 (4H, bs, 3’’-H, 4’’-H). Anal. calcd for [C_27_H_319_FN_2_O_4_+0.2H_2_O]: C, 68.98; H, 6.73; N, 5.96. Found: C, 68.78; H, 6.81; N, 5.54.
