# Supplementary material for: Screening and Development of New Inhibitors of FtsZ from M. Tuberculosis
Source: PLoS One. 2016 Oct 21;11(10):e0164100. doi: 10.1371/journal.pone.0164100 (PMC5074515; doi:10.1371/journal.pone.0164100)
Supplement: S3 Appendix — (DOCX) [file pone.0164100.s003.docx]

**Supporting Information:**

**S3 Appendix. Description of docked poses of a selected set of FtsZ inhibitors.**

**Screening and development of New Inhibitors of FtsZ from *M. tuberculosis***

Bini Mathew,^3^ Judith Varady Hobrath,^4^ Larry Ross,^3^ Michele C. Connelly,^5^ Hava Lofton,^6, 7^ Malini Rajagopalan,^6^ R. Kiplin Guy,^5^ and Robert C. Reynolds^1,2*^

^1^Department of Chemistry, The University of Alabama at Birmingham, Birmingham, Alabama 35294, USA

^2^Division of Hematology and Oncology, The University of Alabama at Birmingham, Birmingham, Alabama 35294, USA

^3^Drug Discovery Division, Southern Research Institute, 2000 Ninth Avenue South, Birmingham, AL 35205, USA

^4^Drug Discovery Unit, College of Life Sciences, University of Dundee, Dundee DD1 5EH, United Kingdom

^5^Dept. Chemical Biology & Therapeutics, St Jude Children's Research Hospital, 262 Danny Thomas Place, Memphis, TN 38105, USA

^6^The University of Texas Health Science Center at Tyler, Tyler, Texas 75708, USA

^7^Current address: Department of Medical Biochemistry and Microbiology, Uppsala University, SE-75123 Uppsala Sweden

**Description of docked poses of a selected set of FtsZ inhibitors.**

Colchicine, Zantrin Z2, AG-825, ChemBridge 5481893, Quercetin were docked into the interdomain cleft site of the *Mtb* FtsZ crystal structure (PDB code 1RLU) using Induced Fit docking protocols. S1 Fig illustrates the obtained poses (except for Colchicine). Hydrogen bonding interactions are illustrated in S1 Fig (dashed lines). In addition to hydrogen bonding, these compounds participate in the following interactions: *Zantrin Z2:* Aromatic – charged interactions include interactions between the phenyl substituent of benzimidazole and the amine of Lys33 and between the benzothiazole and the guanidinium moiety of Arg304, where the center of mass distances between interacting groups are 4.6 Å and 4.7 Å, respectively. Leu188 and Leu299 form non-polar/steric interactions with the benzothiazole while Thr200 and Val294 contribute to favorable non-polar interactions with the ethyl substituent of benzimidazole. Gly31 shows polar interactions with the benzimidazole ring.

*Quercetin:* This ligand forms favorable non-polar interactions with the sidechains of Lys33 and Gln30.

*AG-825:* The ligand participates in aromatic – charged interactions between the benzothiazole moiety and the guanidinium group of Arg304. The sidechains of Gln30, Lys33, Leu188, Thr200 contribute to non-polar and steric interactions.

*ChemBridge 5481893 (Compound* ***12****):* Its oxadiazole-amine group is sandwiched between Arg304 and Gln30. The substituted phenyl shows aromatic – charged interaction with Lys33 (center of mass distance between interacting groups: 4.0 Å).
