# Supplementary material for: Screening and Development of New Inhibitors of FtsZ from M. Tuberculosis
Source: PLoS One. 2016 Oct 21;11(10):e0164100. doi: 10.1371/journal.pone.0164100 (PMC5074515; doi:10.1371/journal.pone.0164100)
Supplement: S4 Appendix — (A) Description and (B) Cartesian coordinates. (DOCX) [file pone.0164100.s004.docx]

**Supporting Information:**

**S4 Appendix. Pharmacophore model.**

**Screening and development of New Inhibitors of FtsZ from *M. tuberculosis***

Bini Mathew,^3^ Judith Varady Hobrath,^4^ Larry Ross,^3^ Michele C. Connelly,^5^ Hava Lofton,^6, 7^ Malini Rajagopalan,^6^ R. Kiplin Guy,^5^ and Robert C. Reynolds^1,2*^

^1^Department of Chemistry, The University of Alabama at Birmingham, Birmingham, Alabama 35294, USA

^2^Division of Hematology and Oncology, The University of Alabama at Birmingham, Birmingham, Alabama 35294, USA

^3^Drug Discovery Division, Southern Research Institute, 2000 Ninth Avenue South, Birmingham, AL 35205, USA

^4^Drug Discovery Unit, College of Life Sciences, University of Dundee, Dundee DD1 5EH, United Kingdom

^5^Dept. Chemical Biology & Therapeutics, St Jude Children's Research Hospital, 262 Danny Thomas Place, Memphis, TN 38105, USA

^6^The University of Texas Health Science Center at Tyler, Tyler, Texas 75708, USA

^7^Current address: Department of Medical Biochemistry and Microbiology, Uppsala University, SE-75123 Uppsala Sweden

**S4A. Description of the pharmacophore model.**

The composite pharmacophore model shown in S2 Fig combines shared pharmacophoric features of five Induced Fit docked FtsZ inhibitors: Colchicine (**1**), Quercetin (**3**) Zantrin Z2 (**8**), ChemBridge 5481893 (**12**) and AG-825 (**15**). This model consists of pharmacophore sites that mark the center of mass of atom groups in these docked poses which form analogous interactions with the same *Mtb* FtsZ residues in closely overlapping regions. S1 Table lists the type of interactions for each pharmacophore site, including contributing FtsZ amino acids and compounds containing the atom groups that participate in these interactions. Eight pharmacophore sites are hydrogen bond donor/acceptor (D/A), one site is hydrogen bond acceptor (A), while the aromatic ring site describes the averaged (center of mass) position of various ring structures. Induced Fit docking protocols allow for side chain flexibility and therefore side chain orientations vary with binding poses. FtsZ residues shown in S2 Fig correspond to coordinates of the FtsZ crystal structure (after structural refinement), which was utilized as input for all docking runs.

**S4B. Cartesian coordinates of the pharmacophore model.**

3-D Cartesian coordinates (PDB format) of the pharmacophore model developed based on the five FtsZ inhibitors (Colchicine, Quercetin, Zantrin Z2, Chembridge 5481893 and AG-825) are given relative to coordinates of residues which interact with or are in proximity to ligand atom groups occupying pharmacophore sites. Coordinates of FtsZ residues are given as present in the FtsZ crystal structure (PDB code 1RLU). The side chain of Lys33 containing missing atoms CE, NZ in the crystal structure was replaced by the closest rotamer from a standard rotamer library. The pharmacophore model consists of the pharmacophore sites 1 – 10, listed as ‘dummy’ residues 1 – 10, respectively, in chain H.

ATOM 1 N GLN A 30 -7.308 41.067 25.224

ATOM 2 CA GLN A 30 -6.933 40.058 26.191

ATOM 3 C GLN A 30 -5.781 40.600 27.018

ATOM 4 O GLN A 30 -5.241 39.901 27.863

ATOM 5 CB GLN A 30 -6.471 38.798 25.493

ATOM 6 CG GLN A 30 -7.579 37.944 24.904

ATOM 7 CD GLN A 30 -8.332 37.240 25.982

ATOM 8 OE1 GLN A 30 -7.792 36.338 26.629

ATOM 9 NE2 GLN A 30 -9.531 37.706 26.256

ATOM 10 N GLY A 31 -5.378 41.828 26.735

ATOM 11 CA GLY A 31 -4.442 42.527 27.594

ATOM 12 C GLY A 31 -2.993 42.165 27.349

ATOM 13 O GLY A 31 -2.226 42.115 28.298

ATOM 14 N LYS A 33 0.565 42.914 27.010

ATOM 15 CA LYS A 33 1.322 44.128 27.411

ATOM 16 C LYS A 33 2.727 44.204 26.846

ATOM 17 O LYS A 33 3.236 43.221 26.289

ATOM 18 CB LYS A 33 1.341 44.219 28.962

ATOM 19 CG LYS A 33 0.038 44.805 29.560

ATOM 20 CD LYS A 33 -0.459 44.108 30.830

ATOM 21 CE LYS A 33 -1.707 44.833 31.350

ATOM 22 NZ LYS A 33 -2.288 44.067 32.467

ATOM 23 N LEU A 188 -2.631 35.470 18.579

ATOM 24 CA LEU A 188 -2.286 36.000 19.890

ATOM 25 C LEU A 188 -1.316 35.067 20.668

ATOM 26 O LEU A 188 -0.341 35.566 21.229

ATOM 27 CB LEU A 188 -3.520 36.309 20.728

ATOM 28 CG LEU A 188 -3.412 36.393 22.286

ATOM 29 CD1 LEU A 188 -2.421 37.492 22.723

ATOM 30 CD2 LEU A 188 -4.796 36.643 22.934

ATOM 31 N ASN A 189 -1.617 33.754 20.741

ATOM 32 CA ASN A 189 -0.804 32.787 21.488

ATOM 33 C ASN A 189 0.638 32.730 20.989

ATOM 34 O ASN A 189 1.543 32.677 21.784

ATOM 35 CB ASN A 189 -1.354 31.357 21.437

ATOM 36 CG ASN A 189 -2.691 31.212 22.122

ATOM 37 OD1 ASN A 189 -3.021 31.921 23.066

ATOM 38 ND2 ASN A 189 -3.483 30.294 21.629

ATOM 39 N GLN A 192 2.284 36.367 22.010

ATOM 40 CA GLN A 192 2.409 36.438 23.461

ATOM 41 C GLN A 192 3.371 35.409 24.104

ATOM 42 O GLN A 192 4.068 35.722 25.085

ATOM 43 CB GLN A 192 1.027 36.338 24.131

ATOM 44 CG GLN A 192 1.082 36.385 25.672

ATOM 45 CD GLN A 192 -0.290 36.651 26.343

ATOM 46 OE1 GLN A 192 -1.346 36.229 25.831

ATOM 47 NE2 GLN A 192 -0.266 37.361 27.465

ATOM 48 N ASP A 196 5.964 37.639 25.988

ATOM 49 CA ASP A 196 5.850 37.668 27.448

ATOM 50 C ASP A 196 7.167 37.163 28.138

ATOM 51 O ASP A 196 7.425 37.525 29.277

ATOM 52 CB ASP A 196 4.655 36.832 27.952

ATOM 53 CG ASP A 196 3.328 37.645 28.065

ATOM 54 OD1 ASP A 196 3.220 38.850 27.652

ATOM 55 OD2 ASP A 196 2.332 37.118 28.579

ATOM 56 N THR A 199 8.817 40.637 28.406

ATOM 57 CA THR A 199 8.199 41.769 29.089

ATOM 58 C THR A 199 7.779 41.400 30.491

ATOM 59 O THR A 199 7.605 42.289 31.310

ATOM 60 CB THR A 199 6.981 42.320 28.315

ATOM 61 OG1 THR A 199 6.002 41.285 28.238

ATOM 62 CG2 THR A 199 7.370 42.630 26.821

ATOM 63 N THR A 200 7.590 40.111 30.773

ATOM 64 CA THR A 200 7.051 39.737 32.098

ATOM 65 C THR A 200 7.583 38.351 32.566

ATOM 66 O THR A 200 6.821 37.427 32.795

ATOM 67 CB THR A 200 5.474 39.928 32.145

ATOM 68 OG1 THR A 200 4.920 39.433 33.391

ATOM 69 CG2 THR A 200 4.790 39.052 31.151

ATOM 70 N VAL A 294 7.053 32.775 32.253

ATOM 71 CA VAL A 294 6.270 33.961 32.237

ATOM 72 C VAL A 294 5.441 34.081 33.529

ATOM 73 O VAL A 294 4.886 33.089 33.987

ATOM 74 CB VAL A 294 5.328 33.925 30.999

ATOM 75 CG1 VAL A 294 4.308 35.088 31.090

ATOM 76 CG2 VAL A 294 6.156 34.021 29.666

ATOM 77 N ASP A 296 2.029 35.452 34.771

ATOM 78 CA ASP A 296 0.718 35.964 34.387

ATOM 79 C ASP A 296 -0.214 35.714 35.578

ATOM 80 O ASP A 296 -0.701 34.625 35.739

ATOM 81 CB ASP A 296 0.239 35.217 33.142

ATOM 82 CG ASP A 296 -1.053 35.791 32.543

ATOM 83 OD1 ASP A 296 -1.711 36.666 33.159

ATOM 84 OD2 ASP A 296 -1.456 35.404 31.421

ATOM 85 N ASP A 297 -0.447 36.724 36.412

ATOM 86 CA ASP A 297 -1.229 36.521 37.640

ATOM 87 C ASP A 297 -2.742 36.319 37.447

ATOM 88 O ASP A 297 -3.458 35.848 38.357

ATOM 89 CB ASP A 297 -0.896 37.597 38.669

ATOM 90 CG ASP A 297 0.233 37.156 39.614

ATOM 91 OD1 ASP A 297 0.168 35.992 40.128

ATOM 92 OD2 ASP A 297 1.222 37.889 39.895

ATOM 93 N SER A 298 -3.211 36.607 36.242

ATOM 94 CA SER A 298 -4.585 36.351 35.853

ATOM 95 C SER A 298 -4.888 34.865 35.568

ATOM 96 O SER A 298 -6.038 34.519 35.274

ATOM 97 CB SER A 298 -4.929 37.189 34.606

ATOM 98 OG SER A 298 -4.604 36.472 33.409

ATOM 99 N LEU A 299 -3.887 33.982 35.635

ATOM 100 CA LEU A 299 -4.096 32.605 35.175

ATOM 101 C LEU A 299 -4.729 31.661 36.229

ATOM 102 O LEU A 299 -5.279 30.592 35.899

ATOM 103 CB LEU A 299 -2.776 32.044 34.642

ATOM 104 CG LEU A 299 -2.378 32.471 33.215

ATOM 105 CD1 LEU A 299 -1.019 31.894 32.836

ATOM 106 CD2 LEU A 299 -3.435 31.996 32.223

ATOM 107 N ARG A 304 0.298 27.574 31.117

ATOM 108 CA ARG A 304 0.168 27.610 29.661

ATOM 109 C ARG A 304 1.547 27.426 29.038

ATOM 110 O ARG A 304 2.471 28.218 29.272

ATOM 111 CB ARG A 304 -0.470 28.946 29.302

ATOM 112 CG ARG A 304 -0.841 29.170 27.828

ATOM 113 CD ARG A 304 -1.456 30.603 27.521

ATOM 114 NE ARG A 304 -0.724 31.717 28.155

ATOM 115 CZ ARG A 304 -1.274 32.637 28.956

ATOM 116 NH1 ARG A 304 -2.588 32.606 29.200

ATOM 117 NH2 ARG A 304 -0.529 33.613 29.492

ATOM 118 CX1 DUM H 1 -1.916 38.788 35.423

ATOM 119 CX2 DUM H 2 -4.400 38.329 30.613

ATOM 120 CX3 DUM H 3 -4.100 34.246 26.409

ATOM 121 CX4 DUM H 4 -2.989 37.222 28.526

ATOM 122 CX5 DUM H 5 1.317 40.367 28.122

ATOM 123 CX6 DUM H 6 3.714 42.062 29.687

ATOM 124 CX7 DUM H 7 1.920 43.124 32.239

ATOM 125 CX8 DUM H 8 -2.315 40.672 31.607

ATOM 126 CX9 DUM H 9 0.443 38.954 36.519

ATOM 127 CAR DUM H 10 -4.919 35.704 28.622

END
