# Supplementary figures and images for: Screening and Development of New Inhibitors of FtsZ from M. Tuberculosis
Source: PLoS One. 2016 Oct 21;11(10):e0164100. doi: 10.1371/journal.pone.0164100 (PMC5074515; doi:10.1371/journal.pone.0164100)

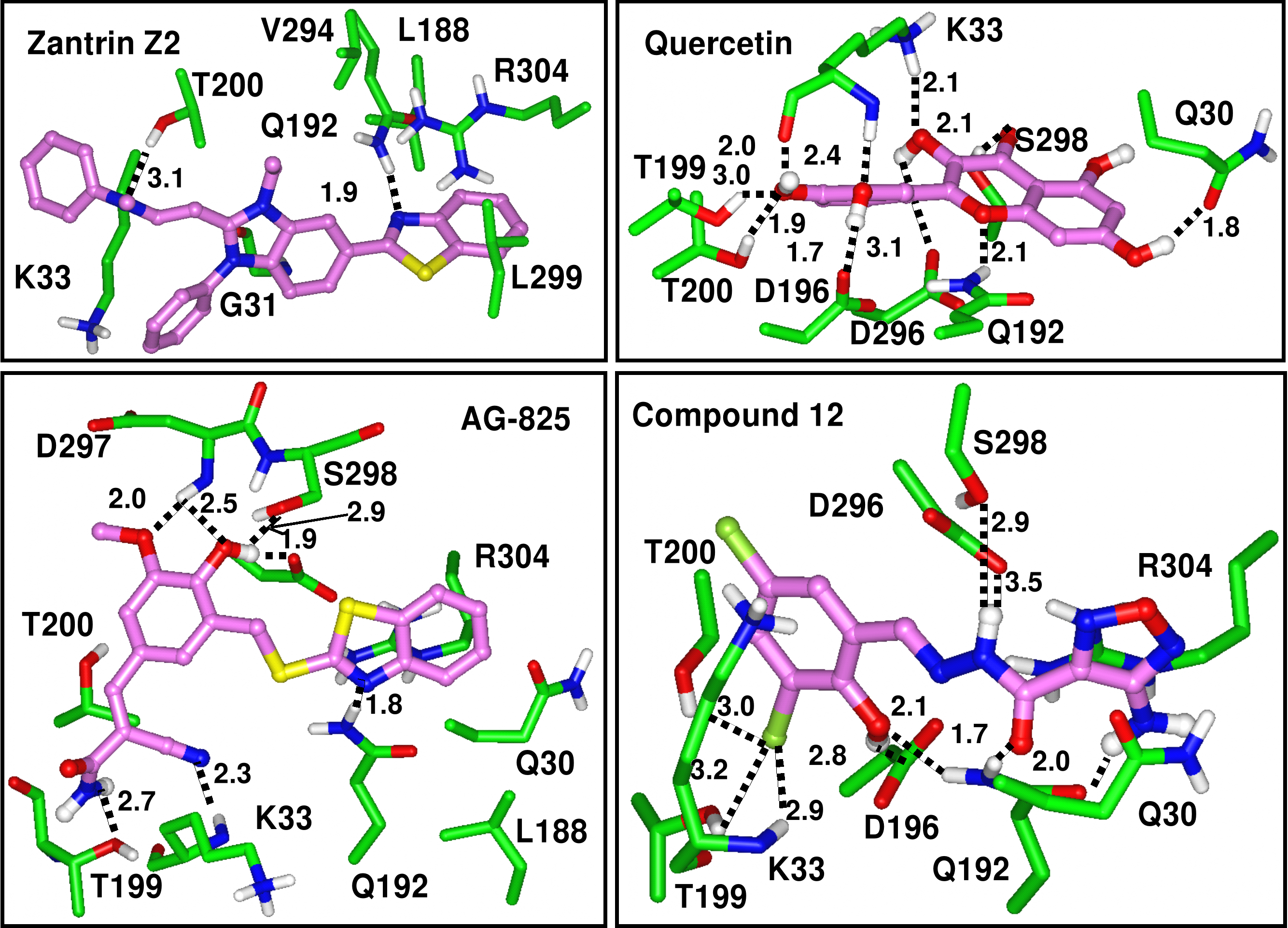

Supplement: S1 Fig — Carbons of the ligand are colored pink, all other atoms by atom type. H-bonds are illustrated with dashed lines. (TIFF) [file pone.0164100.s005.tiff]

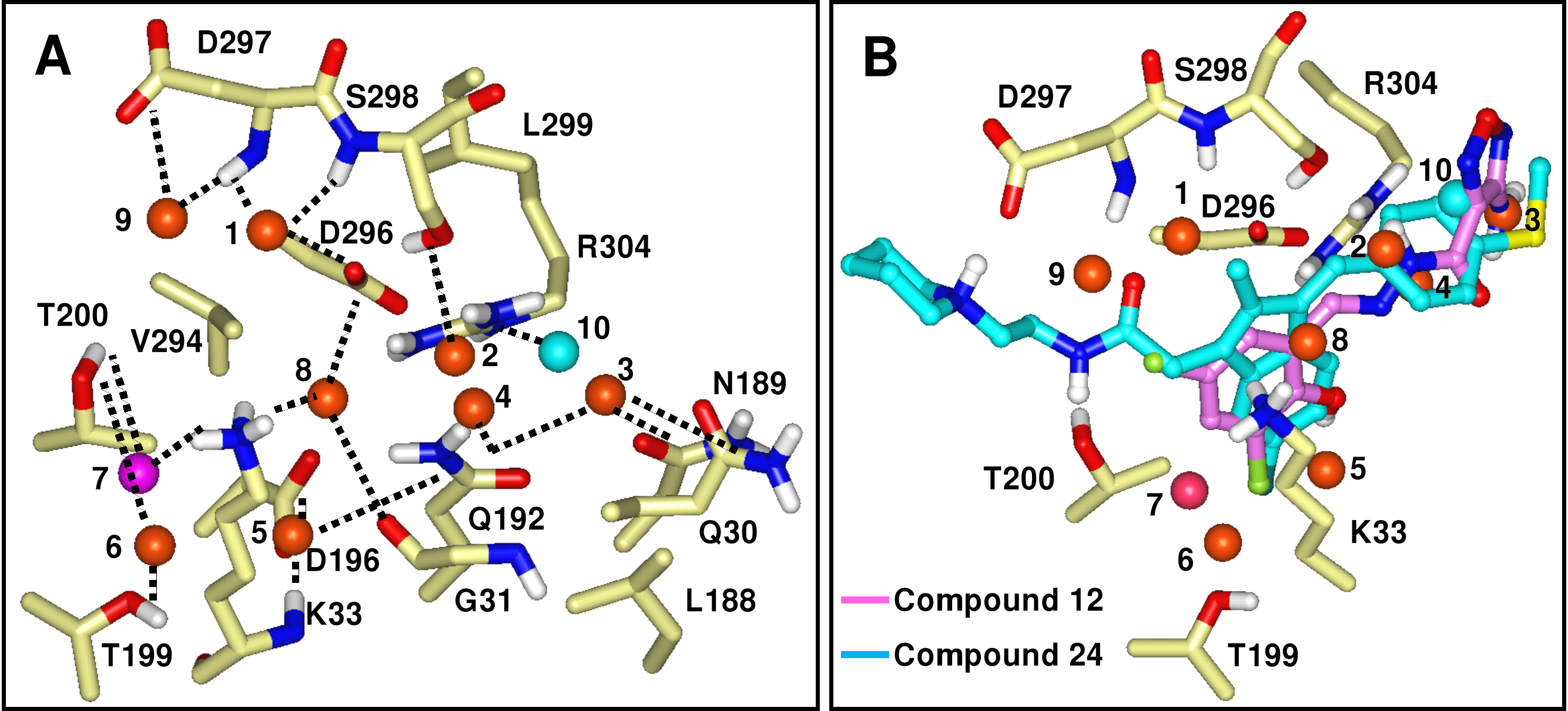

Supplement: S2 Fig — (A) Pharmacophore model sites are shown. Interactions with FtsZ residues are indicated with dashed lines. Hydrogen bond donor/acceptor D/A sites are shown as orange colored spheres, acceptor A site in magenta and aromatic site in cyan. (B) Positions of the docked poses of compounds 12 and 24 in relation to pharmacophoric sites are shown. (TIFF) [file pone.0164100.s006.tiff]
