# Supplementary material for: Screening and Development of New Inhibitors of FtsZ from M. Tuberculosis
Source: PLoS One. 2016 Oct 21;11(10):e0164100. doi: 10.1371/journal.pone.0164100 (PMC5074515; doi:10.1371/journal.pone.0164100)
Supplement: S1 Table — Listed are: type of interactions, Mtb FtsZ residues involved and compounds contributing to each site. Residues interacting through backbone atoms only are marked with (b). (DOCX) [file pone.0164100.s007.docx]

| **Site #** | **Type** | **Residues** | **Compounds contributing** | **Site #** | **Type** | **Residues** | **Compounds contributing** |
| --- | --- | --- | --- | --- | --- | --- | --- |
| *1* | D/A | D297(b), S298(b), D296 | **1**, **15** | *6* | D/A | T199, T200 | **3**, **12**, **15** |
| *2* | D/A | S298 | **3**, **12** | *7* | A | K33, T200 | **1**, **8** |
| *3* | D/A | Q30, N189, Q192 | **3**, **12** | *8* | D/A | G31, K33, D296 | 1, **3**, **8** |
| *4* | D/A | Q192 | **3**, **8**, **12**, **15** | *9* | D/A | D297(b), D297 | **1**, **15** |
| *5* | D/A | K33(b), Q192,D196 | **1**, **3**, **12**, **15** | *10* | Aromatic | R304 | **3**, **8**, **12**, **15** |
